# No-code robotic programming for agile production: A new markerless-approach for multimodal natural interaction in a human-robot collaboration context

**DOI:** 10.3389/frobt.2022.1001955

**Published:** 2022-10-04

**Authors:** Jayanto Halim, Paul Eichler, Sebastian Krusche, Mohamad Bdiwi, Steffen Ihlenfeldt

**Affiliations:** ^1^ Departement of Cognitive Human-Machine System, Fraunhofer Institute for Machine Tools and Forming Technology, Chemnitz, Germany; ^2^ Departement of Production System and Factory Automation, Fraunhofer Institute for Machine Tools and Forming Technology, Chemnitz, Germany

**Keywords:** intuitive robot programming, multimodal intraction, learning from demonstration, human robot collaboration, no-code robotic teaching

## Abstract

Industrial robots and cobots are widely deployed in most industrial sectors. However, robotic programming still needs a lot of time and effort in small batch sizes, and it demands specific expertise and special training, especially when various robotic platforms are required. Actual low-code or no-code robotic programming solutions are exorbitant and meager. This work proposes a novel approach for no-code robotic programming for end-users with adequate or no expertise in industrial robotic. The proposed method ensures intuitive and fast robotic programming by utilizing a finite state machine with three layers of natural interactions based on hand gesture, finger gesture, and voice recognition. The implemented system combines intelligent computer vision and voice control capabilities. Using a vision system, the human could transfer spatial information of a 3D point, lines, and trajectories using hand and finger gestures. The voice recognition system will assist the user in parametrizing robot parameters and interacting with the robot’s state machine. Furthermore, the proposed method will be validated and compared with state-of-the-art “Hand-Guiding” cobot devices within real-world experiments. The results obtained are auspicious, and indicate the capability of this novel approach for real-world deployment in an industrial context.

## 1 Introduction

Human-Robot Collaboration (HRC) has been a prevalent concept in the industry. Compared to the fully automated solution in serial production, HRC offers flexibility to meet the market’s demand for high product variability, diversity, and even batch size 1 as dictated in the current trend of agile production concept (Chryssolouris et al. (2012)). However, reconfiguring and reprogramming the production plan with industrial robots are technical bottlenecks for end-users without or with adequate expertise in robotic programming. Variety and specific domains in robotic programming languages are currently serious impediments to robotic system (re-)deployment in industrial context. Even if an offline programming method is used, refinement in the robot program is required and will cost time until the program is ready to be deployed. An actual survey from state-of-the-art indicated that the lack of HRC know-how, experiences and deployment skills are inhibitors in the deployment of HRC systems. Even though the participants of this survey are *de facto* robotic experts with years experience in the deployment of HRC systems, the results reveal that (re-)configuration of robotic with conventional programming methods is tedious, complex, abstruse and time-consuming (Hornung and Wurll (2022)). Consequently, it triggers a deficiency on productivity and cost efficiency.

Traditionally, robotic programming is categorized in online programming methods, such as traditional lead-trough and walk-trough and offline robotic programming methods, using software tools as the replacement of the real robot system [(Hägele et al. (2016)]. In order to achieve simplification in robotic programming, low- or no-code robotic programming systems are developed. Different novel approaches based on various sensor technologies e.g. 3D tracking system, Augmented Reality (AR), Virtual Reality (VR), Mixed Reality (XR) and motion capture systems, have emerged over the years. Hence, human natural communication modalities substitute prior knowledge of syntaxes and semantics in robotic programming. This concept is known as Programming by Demonstration (PbD) (Billard et al. (2008)) and is also known as Learning from Demonstration (Argall et al. (2009); Lee (2017); Ravichandar et al. (2020)). This approach aims to enable non-robotic experts to teach their robots by demonstrating the desired robots’ behavior or movement in the context of the production process.

Since no expertise to understand a specific robotic programming language is required from the end-user side, robot learning algorithms or strategies are developed to enable the robotic system to understand natural human communication modalities. Thus, it is essential to consider the technological aspects and human-centric issues such as usability and intuitiveness of the interaction between the human and the system. In order to capture, interpret, and understand human instructions accurately and robustly in the context of industrial processes, a novel approach for no-code programming by combining voice and hand gestures is proposed in this work. This combination enables a natural way for humans to interact with the robotic system. As a result, the robotic program can be deployed fast and agile in different industrial scenarios with different robotic systems by applying the proposed architecture in this work. The following section will present an overview of the state-of-the-art. Section 3 will introduce the proposed approach in detail, while section 4 will discuss the implementation of the proposed system. Section 5 will focus on the analysis of the implemented system. Finally, the last section will focus on the conclusion and a short outlook on potential future work.

## 2 Related works

The programming process entails providing a robot with a new ability to understand the state of the environment and perform actions that advance the system towards a process context. Conventionally, the online programming methods use a teach pendant to move a robot through the desired motion profile by jogging. The robot movement is stored in the robot controller and can be retrieved later. Even though the method seems to be simple and demands less expertise, online programming is suitable for simple repetitive tasks, e.g. industrial processes with simple movement profiles and geometric workpieces. When changes occur, adaptation to the robotic program is required. Hence, this approach is only suitable for production with large lot sizes. The frequent reconfiguration is tedious, unaffordable and time-consuming for small and medium enterprises with smaller batch sizes (Dietz et al. (2012)).

Offline robotic programming methods are deployed to replace the online robotic programming methods (Neto and Mendes (2013)). In offline programming methods, a virtual environment representing the robot work cell is created to program the robot’s behaviour and motion. The robot programmer can generate a robot program off-site *via* offline programming methods. Hence production downtime can be avoided during the programming phase. Extendable functions for robotic programming, e.g. path planning and control system for complex production processes, are embedded in most offline programming tools (Beck et al. (2021); Funes-Lora et al. (2021)). A virtual robot controller (VRC) simulates the exact robot behaviour for a specific robot platform in the virtual environment. In many cases, the virtual environment mismatches the environment. For high-precision applications, adjustments in the robotic program must be performed to eliminate the deviations in transferring the robot program to the actual robot controller (Angelidis and Vosniakos (2014)).

With the rise of collaborative robots, the perspective of robotic programming shifted in the last decade. Safety and ease of use are crucial factors in developing collaborative robot systems. In many collaborative robot systems, hand-guiding control methods are deployed to accelerate robotic teaching compared to traditional methods (Massa et al. (2015)). In the PbD context, teaching *via* hand-guiding control is used to demonstrate the robot behaviour using a kinesthetic teaching process. Hand-guiding control is specified in actual standards of industrial robotic systems (DIN ISO/TS 15066 (2017); DIN EN ISO 10218-1 (2021); DIN EN ISO 10218-2 (2012)). In recent years, hand-guiding controls have been implemented in many industrial applications, e.g. robotic gluing (Iturrate et al. (2021)), assembly (Liu et al. (2021)), polishing (Kana et al. (2021)), welding (Zhang et al. (2019)), surface cleaning (Elliott et al. (2017)), Pick-and-Place or manipulation (Peng et al. (2018)). Despite the ease of hand-guiding teaching methods, these hand-guiding demands medium to high physical workload to move the robot joints. To improve users’ ergonomics, algorithms, e.g. gravity compensation and variable stiffness, are developed to reduce the workload in kinesthetic teaching (Infante and Kyrki (2011); Wrede et al. (2013); Tykal et al. (2016)). The compensation algorithms mentioned above utilize dynamic parameters of the robotic system. In the implementation, this information is inaccessible to the robot manufacturers. The accuracy of the taught robotic path *via* kinesthetic teaching depends on the dexterity of the end-user. Hand tremor and lack of force in programming affect the quality and the precision of the robot path (Massa et al. (2015)). In order to compromise the physical workload in the kinesthetic teaching process, the teleoperation concepts are introduced where the users can manipulate the robot in real-time by using their gestures or body movements. In general, the teleoperation approaches are performed by utilizing different type of haptic sensors such as mid-air haptic devices (Du and Zhang (2014)), electroencephalograms (EEGs) (Yang et al. (2018a)) and joysticks (Sanchez-Diaz et al. (2019)).

Strategies such as teleoperation, observation and imitation are used to transfer human knowledge into robotic platforms. Vision-based systems, speech recognition systems, AR, VR and XR technologies are developed to accelerate low-code or no-code robotic programming methods (El Zaatari et al. (2019); Villani et al. (2018)). In low-code programming methods, adequate know-how in a robot programming language is still required. As a result, the reconfiguration of the robot program is time consuming. Compared to low-code programming, no-code robotic programming eliminates the barriers by allowing the user to interact with or move the robot using natural interactions, e.g., voice, gesture or haptic. In recent works from state-of-the-art, vision-based systems are exploited in many intuitive programming methods due to the capabilities of vision systems in environment recognition, object recognition and gesture recognition. In (Zhang et al. (2020c)), a novel approach for robot path teaching is developed using a marker-based vision system with a single RGB-D camera. The movement of the marker is tracked with the RGB-D camera and transferred into a motion planner. In the recent works (van Delden et al. (2012); Akkaladevi et al. (2019, 2020); Ajaykumar et al. (2021)), several works address intuitive programming approaches *via* vision systems for specific processes such as Pick-and-Place and assembly. In (van Delden et al. (2012)), a multimodal teaching approach *via* gesture and voice is developed for the Pick-and-Place application. This approach allows the user to select the objects and target position for the manipulation process by using a deictic finger gesture. Hence, a voice command is given to the robot to pick or place the object. An intuitive programming approach by demonstration is developed in (Akkaladevi et al. (2020)). This approach uses a multi-camera setup to track the assembly tasks performed by the user. The human actions and assembly objects will be tracked and used to build a knowledge representation of the assembly tasks, which will be sent to the robot system. In (Ajaykumar et al. (2021)), a marker-based programming strategy is developed by using objects with markers for the Pick-and-Place scenario. The robot path is created by manipulating the objects. The object movement will be tracked and converted as a robot program.

The emergence of AR/XR/VR technologies has influenced the programming strategies in HRC. In Akkaladevi et al. (2019), lighthouse sensors are used to demonstrate the user movement in a complex assembly process with screwing actions. A programming device is created by combining the lighthouse sensors for spatial tracking and force and torque sensors to measure the required torques for the screwing process. A combination of a vision-based system with augmented reality technology is introduced in (Lambrecht et al. (2013)). The augmented reality system allowed the teaching of robot paths by manipulating spatial objects with hand gestures. Other approaches with augmented reality technology are developed in (Soares et al. (2021); Blankemeyer et al. (2018); Bolano et al. (2020)). In (Soares et al. (2021)), a Microsoft HoloLens 2 1 is to develop an augmented reality environment. This environment enables the users to interact with the robot by drawing the robot path with their fingers. Afterwards the teaching process, the robot path is transferred into the robot system. In (Blankemeyer et al. (2018)), an intuitive programming approach for the assembly process is performed in an augmented reality environment. A representation of the assembled object is built in the virtual environment and the assembly process with the virtual object is demonstrated. Hence, this information will be transferred to the robot to execute the assembly task. In (Bolano et al. (2020)), an offline programming method in a virtual reality environment is developed. The robot trajectory can be generated by manipulating the virtual robot. Hence, the trajectory will be sent to a graphic interface to be executed in a real robot. *Via* the graphic interface, the movement sequence can be configured.

Besides using one modality to perform intuitive robot programming, more interactions can be used to increase the acceptance and comprehensibility of the teaching process. In (Liu et al. (2020)), a programming approach with the combination of sensorless haptic interaction, voice instructions, and hand gesture commands is used in an assembly scenario. The voice system helps the user to move the robot TCP. The hand gesture can perform the fine adjustment of the robot’s position. Hence, the defined function blocks for the assembly and manipulation system can be triggered *via* voice instructions. In (Tirmizi et al. (2019)), a multimodal programming approach with a voice and vision system is developed for the Pick-and-Place scenario. The voice recognition system is utilized to control the system state. A vision-based object recognition system tracks the objects and delivers their coordinates that can be used for the manipulation process. In (Strazdas et al. (2022)), a multimodal system with a gesture, speech and gaze recognition system is developed for the Pick-and-Place scenario. The face and gaze recognition system monitors the interaction context with the system. The voice recognition system is used to control the robot’s state. *Via* deictic gestures, the interaction objects can be chosen. In the recent multimodal programming approaches, a voice recognition system is integrated to navigate and control the system state. A recent study proved that a voice input system could accelerate robot programming up to two times in comparison to using traditional input devices (e.g., keyboards, teach pendants) (Ionescu and Schlund (2021)).

## 3 Methods

### 3.1 Proposed architecture

#### 3.1.1 System architecture

**FIGURE 1:**
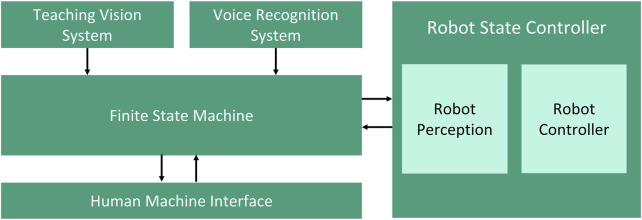
Proposed system architecture for multimodal no-code robotic programming.

The proposed system architecture consists of five modules which are depicted in Figure 1. The modular system design allows each functionality to be encapsulated as a subsystem. As a result, the highest degree of flexibility can be achieved in the system. The modular system architecture allows a better comprehensibility of the source codes, the simplification of the problem solving and the fast integration of new functionalities (Zirkelbach et al. (2019)).

A combination of hand- and finger-gestures with speech is proposed in the system architecture to allow a natural interaction in the teaching process of the robotic system. In comparison to low-code programming, no-code robotic programming method *via* multimodal interaction allows the user to create a robot program without particular expertise in robotic programming language. The robot program can be (re-)configured just by using interaction modalities that human does to communicate with each other. In this work, the proposed no-code programming is implemented by recognizing the hand- and finger-gestures *via* teaching vision system and recognizing user input *via* voice in the speech recognition system.

A camera-based vision system is developed to track and recognize the user’s hand- and finger gestures in the teaching phase. The coordinates of the hand- and finger gestures are tracked and processed with computer vision algorithms to estimate the spatial pose in defined coordinate system. The coordinates of the hand or finger will be recorded based on the given commands and will be used to generate a robot path after the teaching process. This information will be converted into a specific robotic programming language before being transfer into the robotic system. The robotic system is equipped with a camera system as a perception module for executing the given robot path. Camera systems are considered in the proposed approach due to their benefits in comparison to other motion capture technologies such as (e.g: IMU- and VR systems). In general camera systems are markerless, easy to use, easy to set up, and affordable. In recent years, many reliable algorithms have been developed and shown potential to improve the camera system’s performance, even compensating for their drawbacks (El Zaatari et al. (2019)).

The voice recognition system works as a complement to the teaching vision system to configure the system states and parameters. In this work, the speech recognition system will process the user voice into text *via* Text-To Speech (TTS). Hence, the articulation of the voice command will trigger a deterministic action in the finite state machine. When a user says “take point,” the actual coordinate of the finger will be extracted in the robot path. *Via* voice recognition system, efficiency in robotics programming is achieved by eliminating unnecessary user interactions *via* traditional human-machine interfaces (HMIs), e.g. buttons, keyboards, and mouse clicks. A recent study showed the potential of a speech recognition system to improve time efficiency in human-computer interface up to three times (Ruan et al. (2016)). A graphical HMI is developed to give the user visual feedback of the system. The HMI can be used as a redundant input system when the speech recognition system fails due to transient environmental noises.

#### 3.1.2 System requirement

The system requirements for the proposed approach are depicted in Tables 5, 6. These system requirements must be fulfilled to enable fluent, stable and satisfactory interactions in the proposed robotic teaching process.

### 3.2 Teaching vision system

**FIGURE 2:**
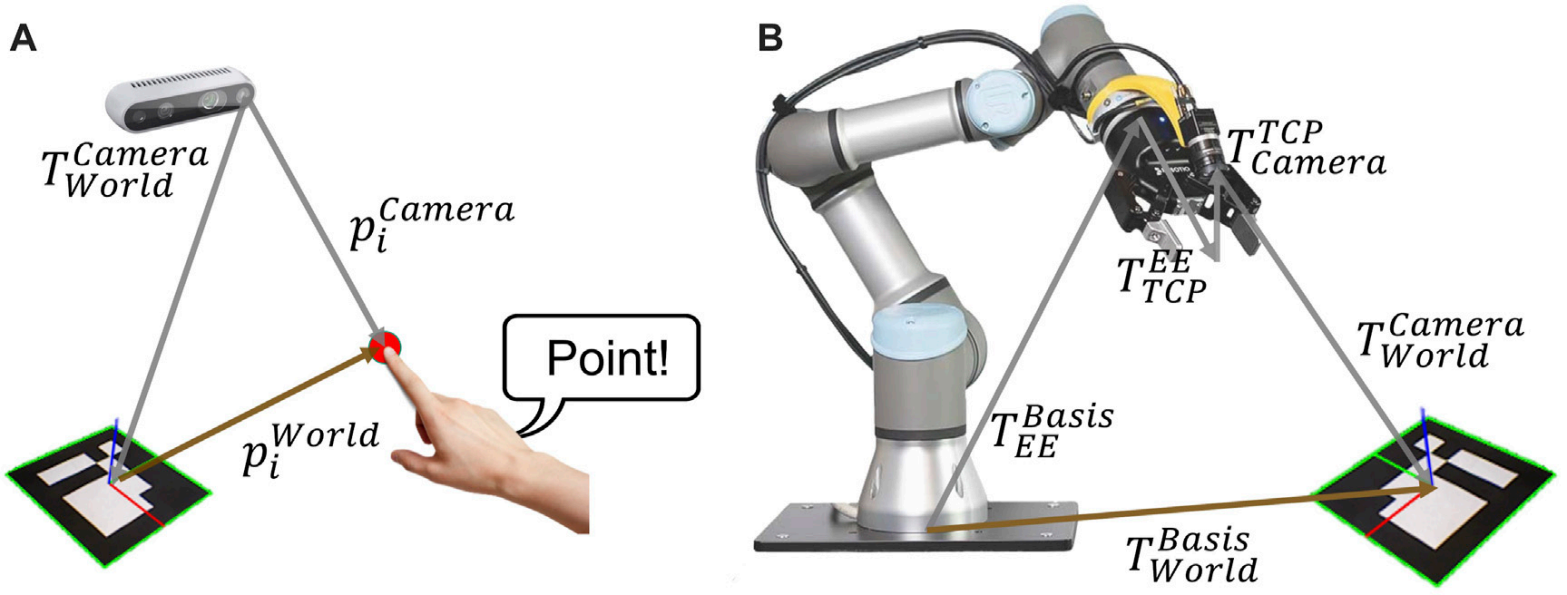
**(A)** transformation chain for the *i*th point of the robot path from programming process related to the target coordinate, **(B)** transformation chain for robotic perception system from robot base to target coordinate system.

A vision-based teaching system is proposed for the main interaction modality of the novel teaching method. In Figure 2, the transformation chain for the programming process and robotic perception system are shown. For the proposed programming method, the world or target coordinate system is implemented by using an ArUco marker (Garrido-Jurado et al. (2014)). In comparison to other fiducial markers, e.g. ARTag, STag. ArUco marker guarantees high-precision position detection even in the noisy environments and utilizes low-computational power (Zakiev et al. (2020); Kalaitzakis et al. (2020)).

Figure 2A shows the transformation chain of the actual index finger’s coordinates in the teaching process. The finger coordinates are captured from the camera system in the pixel coordinates. Hence, the finger coordinates are transformed in Cartesian coordinate with respect to the target coordinate system by using direct linear transformation. As a result, the target coordinate $p_{i}^{\mathrm{Target}}$ can be expressed with Eq. 1.

$$p_{i}^{\mathrm{Target}}={\left(T_{\mathrm{Target}}^{\mathrm{Camera}}\right)}^{-1}p_{i}^{\mathrm{Camera}}$$ (1)

Figure 2B shows the transformation chain for the homogenous transformation from base to target coordinate system $T_{\mathrm{Base}}^{\mathrm{Target}}$ for the robot path. This transformation chain can be mathematically formulated using the equation in (2) and will be discussed in 3.2.1.3.

$$T_{\mathrm{Base}}^{\mathrm{Target}}=T_{\mathrm{Base}}^{EE}T_{EE}^{\mathrm{TCP}}T_{\mathrm{TCP}}^{\mathrm{Camera}}T_{\mathrm{Camera}}^{\mathrm{Target}}$$ (2)

#### 3.2.1 Hand- and finger-gesture recognition system

##### 3.2.1.1 Hand- and finger-tracking

From the state-of-the-art, machine learning based hand- and finger-tracking SDKs are MediaPipe (Zhang et al. (2020b)), OpenPose (Simon et al. (2017)), AWR for hand 3d pose (Huang et al. (2020)) and MMPose (MMPose-Contributors (2020)). The mentioned SDKs allow hand- and finger-tracking by using RGB-image as input. Compared to the traditional computer vision-based algorithms, machine learning-based hand- and finger-tracking algorithms deliver better performance tracking under different lighting conditions, reflections, skin colours, and transitions over background objects with colour as human skin. The traditional computer vision tracking algorithm generally converts the input RGB image into another colour space. Classification is performed by defining the tracking colour constraints concerning the tracked object characteristics. As a result, unexpected objects will not be recognized. For example, a hand-gesture recognition system based on HSV colour space was implemented for an automatic handing-over system between heavy-duty and human co-workers (Bdiwi et al. (2013b)). This computer vision-based algorithm showed limits when tracking hand over reflective objects or objects with colour as human skin.

The main essential aspects for choosing the hand- and finger tracking SDK are the tracking performance based on the frame rate (FPS) and robustness under different light conditions. Besides, the specific hand model and its key points (landmarks) are considered for this proposed method. In experiments, MediaPipe constantly delivered 30 FPS with CPU computing. On the other hand, OpenPose delivered only 5 FPS with CPU computing. Even though the 2× up to 3× frame rate can be reached using GPU, it was not sufficient to provide fluent interaction for the proposed method. MediaPipe utilizes a hand model with 21 key points as shown in Figure 13. The index finger’s tip (landmark 8) is tracked and used as a reference for the position in the teaching process. The finger’s orientation is derived by calculating a Rodrigues vector between two landmarks in the index finger (landmarks 8 and 7). As a result, a robot path can be created by drawing splines or depicting singular points in the teaching process. It should be taken into account that the inaccuracies of the finger orientation calculation can occur due to the camera’s limited field of view and perspective.

##### 3.2.1.2 Pose estimation of the finger landmark

Assuming that the camera is a pinhole model, a direct linear transformation is used to obtain a projection of a point of interest in the target coordinate system (3D) into the pixel coordinate system (2D) or vice versa. Eq. 4 describes the transformation for rectified image. In this equation, *s* is the scaling factor, *u* and *v* are the coordinates of a point of interest in pixel coordinate. The intrinsic parameters of the camera are characterized by *f*_*x*_, *f*_*y*_, *c*_*x*_, and *c*_*y*_. *f*_*x*_ and *f*_*y*_ are the x- and *y*-axis focal length of the camera in a pixel unit. *c*_*x*_, and *c*_*y*_ are the x- and *y*-axis optical center of the camera in a pixel unit. *X*_*c*_, *Y*_*c*_ and *Z*_*c*_ are the coordinates of the point of interest in the camera coordinate system. By using a homogenous transformation matrix between the camera and target $T_{\mathrm{Target}\left(4x4\right)}^{\mathrm{Camera}}$ , the coordinates of the point of interest in the camera coordinate system are decomposed into coordinate points in the target coordinate system (*X*_*w*_, *Y*_*w*_ and *Z*_*w*_). The transformation matrix between camera and target is mathematically formulated with Eq. 3.

$$T_{\mathrm{Target}\left(4x4\right)}^{\mathrm{Camera}}=\left[R_{\mathrm{Target}\left(3x3\right)}^{\mathrm{Camera}}\|t_{\mathrm{Target}\left(3x3\right)}^{\mathrm{Camera}}\right]$$ (3)

with $R_{\mathrm{Target}\left(3x3\right)}^{\mathrm{Camera}}$ the rotation matrix and $t_{\mathrm{Target}\left(3x1\right)}^{\mathrm{Camera}}$ the translation vector. The rotation matrix and translation vector represent the extrensic parameters of the camera.

The target coordinate system in this teaching process is represented by ArUco marker. All the points taken in the robot path will be transformed into the target coordinate system. In general, the 3D-coordinate points of the landmark (finger) relative to the ArUco marker is calculated by solving (4) in target coordinate points. Assuming that the finger is moving in different planes in 3D, the scaling factor *s* in (4) is varied according to the current plane parallel to the camera sensor. Hence, *s* is equal to the depth information of the finger in the camera coordinate system *z*_*finger*_. This information can be derived directly from the depth image of the camera. The spatial information of the finger on x- and *y*-axis of the camera coordinate are calculated by using the intrinsic parameters *f*_*x*_, *f*_*y*_, *c*_*x*_ and *c*_*y*_ as shown in (5). Since diagonal elements of the transformation matrix between camera and target $R_{\mathrm{Target}\left(3x3\right)}^{Camera^{-1}}$ is always not equal to zero the inverse of this matrix can be performed normally.

$$s\left[\begin{array}{c} u\\ v\\ 1 \end{array}\right]=\left[\begin{array}{ccc} f_{x} & 0 & c_{x}\\ 0 & f_{y} & c_{y}\\ 0 & 0 & 1 \end{array}\right]\left[\begin{array}{c} X_{c}\\ Y_{c}\\ Z_{c} \end{array}\right]=\left[\begin{array}{ccc} f_{x} & 0 & c_{x}\\ 0 & f_{y} & c_{y}\\ 0 & 0 & 1 \end{array}\right]\left[R_{\mathrm{Target}\left(3x3\right)}^{\mathrm{Camera}}\|t_{\mathrm{Target}\left(3x1\right)}^{\mathrm{Camera}}\right]\left[\begin{array}{c} X_{w}\\ Y_{w}\\ Z_{w}\\ 1 \end{array}\right]$$ (4)

$$\left[\begin{array}{c} X_{w}\\ Y_{w}\\ Z_{w} \end{array}\right]=R_{\mathrm{Target}\left(3x3\right)}^{Camera^{-1}}\left(\begin{array}{c} \left[\begin{array}{c} \frac{u_{\mathrm{finger}}-c_{x}}{f_{x}}z_{\mathrm{finger}}\\ \frac{v_{\mathrm{finger}}-c_{y}}{f_{y}}z_{\mathrm{finger}}\\ z_{\mathrm{finger}} \end{array}\right]-t_{\mathrm{Target}\left(3x1\right)}^{\mathrm{Camera}} \end{array}\right)$$ (5)

In this work, the camera image is already rectified and the intrinsic parameters are accessible from the SDK of the camera. Otherwise intrinsic calibration can be performed by using function in OpenCV (Qiao et al. (2013)) or another tool like MATLAB. The rotation matrix and translation vector with respect to the marker is calculated *via* extrinsic calibration. The calculation of the rotation matrix and translation vector can be performed by using Perspective-n-Point (PnP) pose computation using approach (Marchand et al. (2016)) or OpenCV function for estimating pose of the single ArUco marker.

##### 3.2.1.3 Image processing of spatial information of the finger landmark

**FIGURE 3:**
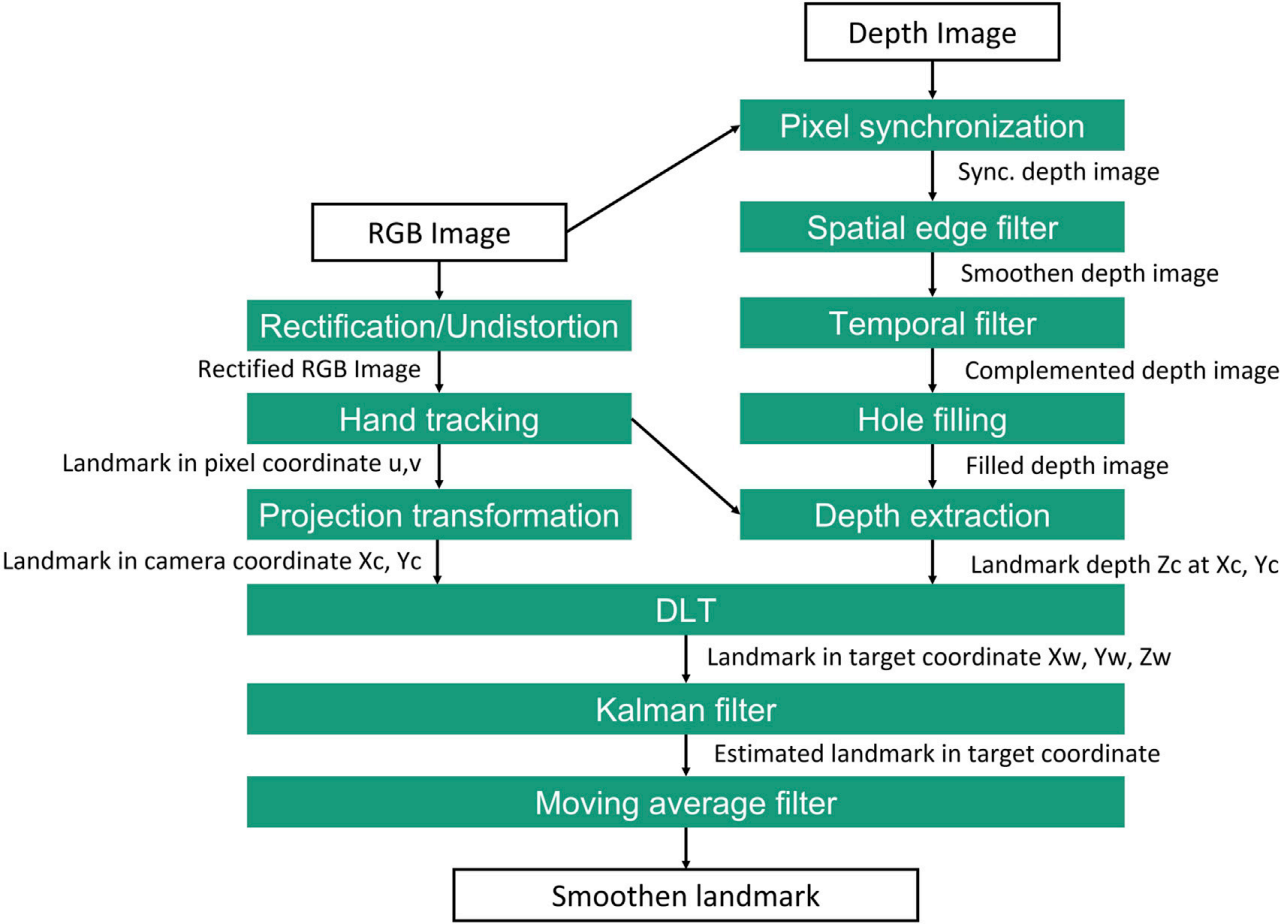
Proposed image processing method for extraction 3D coordinate of landmark for programming process.

With the advent of the computer vision algorithm, significant improvements in the accuracy of the teaching system can be achieved by implementing proposed algorithms, which are shown in Figure 3. Since the resolution of the RGB and depth image are not the same, it is necessary to synchronize the depth image with the RGB image. Hence, the RGB image is rectified to correct the distortion in the image. The depth image processing is executed in parallel to the RGB-image processing. The spatial edge filter is used to enhance the smoothness of the depth reconstructed data by performing a series of 1D horizontal and vertical passes or iterations (Gastal and Oliveira (2011)).

A temporal filter is implemented to add the missing depth information when the pixel unit is missing or invalid. The data is processed in a single pass to adjust the depth values based on previous frames in this procedure. Hence, a hole-filling filter can fill the missing depth values using iteration based on the nearest pixel neighbours (Cho et al. (2020)). In the following step, the hand tracking method described in 3.2.1.1 is performed to obtain pixel coordinate *u*, *v* of the finger landmark. Simultaneously the transformation of the pixel coordinate into camera coordinate *X*_*c*_, *Y*_*c*_, and depth information *Z*_*c*_ extraction for the respected pixel unit of the finger landmark are performed. Then the landmark coordinate based on camera is fused and transformed into target coordinate *X*_*w*_, *Y*_*w*_ and *Z*_*w*_ by using (5). Since the frame rate of the tracking system is limited to 30 FPS, stable hand tracking may not be available due to the fast movement of the hand. Therefore a Kalman filter is used to estimate the landmark position when tracking is missing or invalid in a short period. The kalman filter function from the OpenCV is utilized in this work. Finally, a moving average filter is implemented to smoothen the landmark position. The window size should be parameterized so that the filter does not cause any frame rate loss.

### 3.3 Voice recognition system

As already mentioned in 3.1.1, the voice recognition system is used to assist the end-user in changing the system state and parameter. The end user’s speech commands are extracted as text *via* Text-To Speech (TTS). After the feature extraction, the text is matched and proved with Natural Language Understanding (NLU) algorithm. In comparison to the traditional voice recognition system, NLU-based voice recognition system can deliver better performance and eliminate outliers with different voice characteristics (e.g., accents and voice profiles). In traditional voice recognition systems, the recognizer is built based on three models: *1*) acoustic models represent the acoustic signals of the voice, *2*) language models represent the grammars and semantics of the languages, *3*) lexicon models represent the phonemes and phonetics of word (Karpagavalli and Chandra (2016)). These models must be developed manually and it is impossible to create a general model that can cover heterogeneous voice profiles of the speakers. NLU-based voice recognition systems use deep learning models based on trained data sets. With this approach, a better performance and more generic solution for voice recognition can be achieved.

### 3.4 Robot state controller

The robot state controller controls the behavior of the robot after receiving the generated robot path from the teaching process. The robot path from the teaching process is transformed to target coordinate system. The robot controller takes Cartesian coordinates at the robot base as reference for the robot movement. Therefore a coordinate transformation between the robot base and the target is performed With the assistance of a vision-based perception system.

It is sufficient to use the perception system to detect the target and apply the transformation with the target as the reference coordinate system for the robot. In other words, the robot movement is executed relative to the marker after the coordinate system transformation is performed. The transformation problem of the robot trajectory between robot base coordinate system and target coordinate system is accomplished by solving the equation of the transformation chain in (6).

$$T_{\mathrm{Base}}^{\mathrm{Target}}=T_{\mathrm{Base}}^{EE}T_{EE}^{\mathrm{TCP}}T_{\mathrm{TCP}}^{\mathrm{Camera}}T_{\mathrm{Camera}}^{\mathrm{Target}}$$ (6)

**FIGURE 4:**
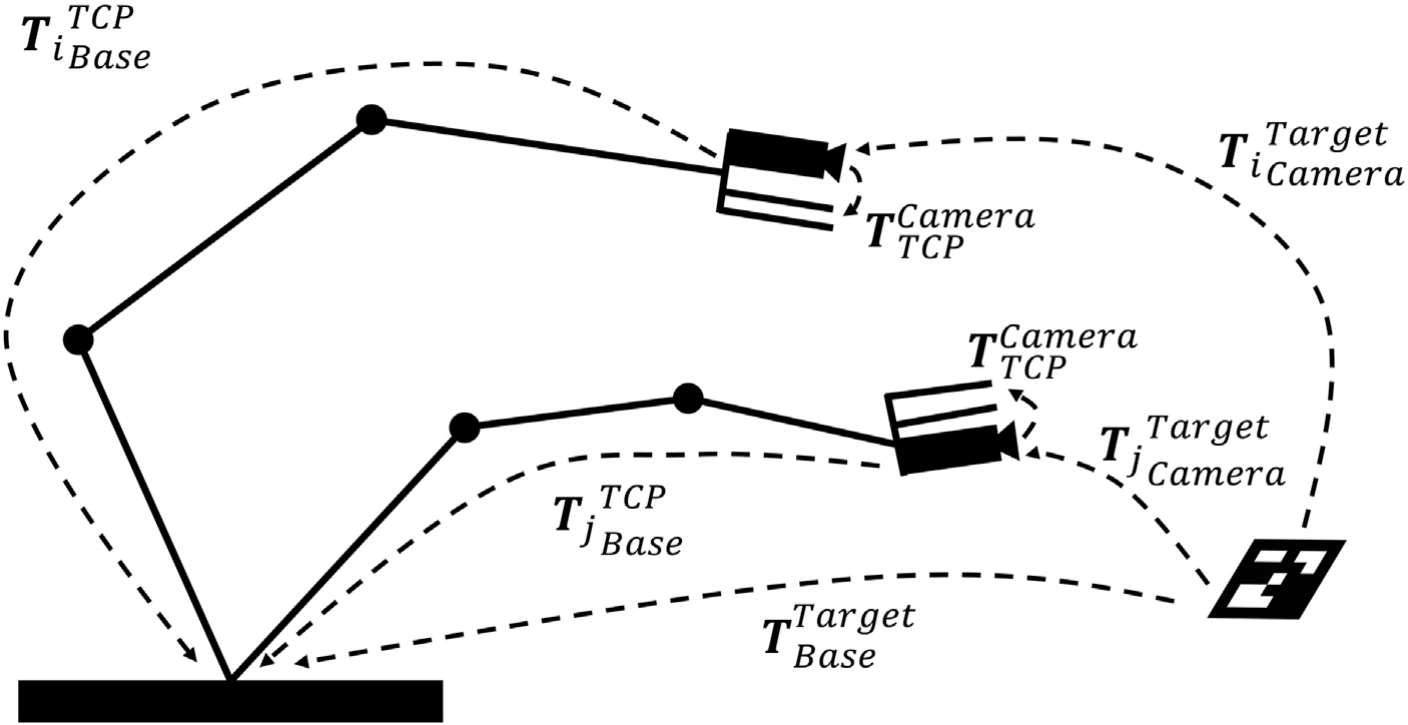
Hand-eye calibration problem: solving $T_{\mathrm{Camera}}^{\mathrm{TCP}}$ using relative TCP and camera movements.

The homogeneous transformation matrix from Base to EE $T_{\mathrm{Base}}^{EE}$ and transformation matrix from EE to TCP $T_{\mathrm{Base}}^{EE}$ is determined known by converting the TCP position from the robot interface into a 4 × 4 matrix. In order to obtain the transformation between the camera and TCP $T_{\mathrm{TCP}}^{\mathrm{Camera}}$ the hand-eye calibration problem has to be solved by moving the robot into several positions. The resulting movements of the eye (camera) are observed as shown in Figure 4.

At this moment, the transformation matrix between the base and target $T_{\mathrm{Base}}^{\mathrm{Target}}$ should be equal in each relative movement of the robot as mathematically formulated in (7).

$$T_{iBase}^{\mathrm{TCP}}T_{\mathrm{TCP}}^{\mathrm{Camera}}T_{iCamera}^{\mathrm{Target}}=T_{jBase}^{\mathrm{TCP}}T_{\mathrm{TCP}}^{\mathrm{Camera}}T_{jCamera}^{\mathrm{Target}}$$ (7)

By converting the (7) into (8), the transformation matrix of the target to the camera $T_{\mathrm{Camera}}^{\mathrm{Target}}$ can be obtained using the pose estimating method (PnP) as described in 3.2.1.2.

$${\left(T_{jBase}^{\mathrm{TCP}}\right)}^{-1}T_{jBase}^{\mathrm{TCP}}T_{\mathrm{TCP}}^{\mathrm{Camera}}=T_{\mathrm{TCP}}^{\mathrm{Camera}}T_{jCamera}^{\mathrm{Target}}{\left(T_{iCamera}^{\mathrm{Target}}\right)}^{-1}$$ (8)

In this work, numerical approach provided in OpenCV function is used to solve the hand-eye calibration problem. OpenCV provides five different calibration methods that differ in the order in which orientation and translation are estimated. In the following they will named after their authors and in line with the OpenCV documentation : Tsai (Tsai and Lenz (1989)), Park (Park and Martin (1994)), Horaud (Horaud and Dornaika (1995)), Andreff (Andreff et al. (1999)) and Daniilidis (Daniilidis (1999)). The results of our hand-eye calibration by applying the five mentioned algorithms above were converged. It means that the algorithms delivered the same results with minor offsets from each other.

### 3.5 Finite state machine

The finite state machine works as the main controller of the system. The speech commands are used as transition signals for the state machine. As a result, a deterministic action will be executed depending on the defined states in the state machine. Explicitly, the implementation of the finite state machine will be discussed more in detail in 4.3.

### 3.6 Human machine interface

To provide the user with feedback, a graphical user interface (GUI) was implemented. Information such as videos from the teaching and robot perception vision system, given speech commands, system parameters and statuses is represented in the GUI. The user interface serves not only as feedback, but also as a redundant input system. This is intended, for example, when the speech recognition system is not usable due to too intense ambient noise. Actual research showed that the relevance of user interfaces in hybrid human-robot systems can improve user acceptance and reduce mental workload (Bdiwi et al. (2021)).

## 4 Implementations

### 4.1 Setup

**FIGURE 5:**
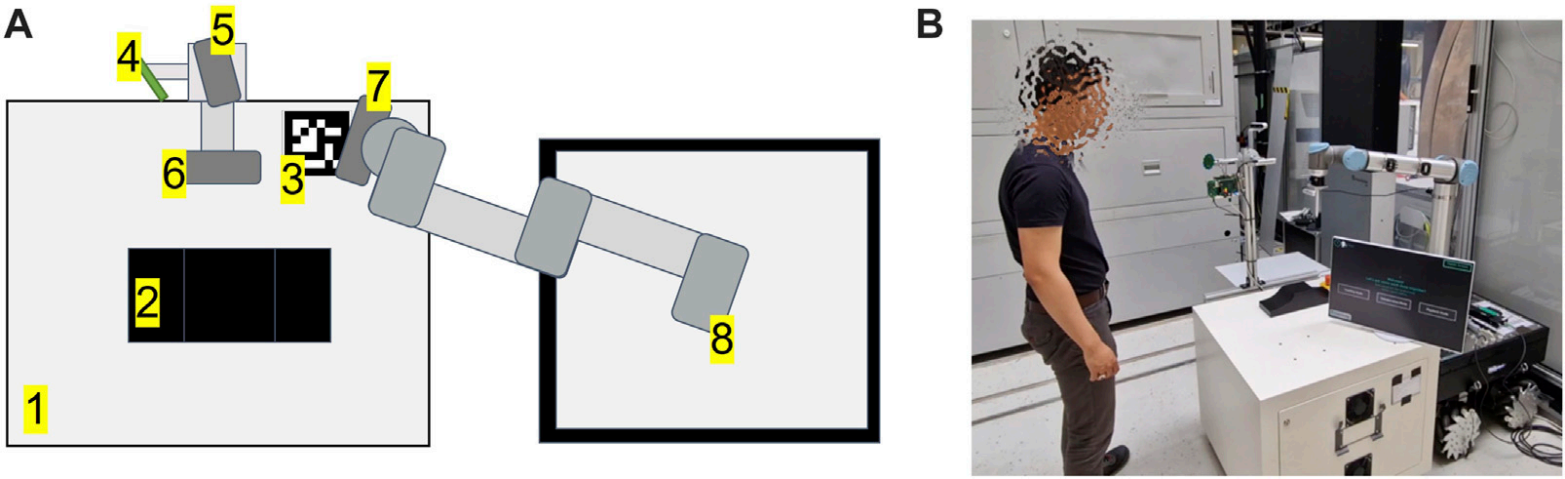
**(A)** setup of the proposed system. From 1 to 8: working table, workpiece, ArUco marker, Microphone array, Intel RealSense D415 - parallel to working table, Intel RealSense D435 - frontal to the user, Intel RealSense D415 - robot vision, Universal Robot UR10 CB3-Series, **(B)** setup in the real world.

Figure 5 shows the experimental setup for the proposed multimodal programming approach in this work.

The hardware used in this setup has been fulfilled the system requirements suggested in Appendix I - system requirements. An Universal Robot UR10 CB-Series is used as the robotic platform (Robots (2015)). UR RTDE 2 is used as communication interface between an industrial PC and the UR10. Three Intel RealSense D400 Series cameras are used for the interaction process (Intel (2015)). One Intel RealSense D415 camera is placed parallel to the surface of the working table is used to capture the spatial information of the gesture during the teaching process, as mentioned in 3.2.1. The camera is located 64 cm above the table surface, delivering a 48 cm × 32 cm field of view. Since the field of view has linear correlations with camera height, putting the camera at a higher height would increase the field of view. All of the camera positioning is flexible and can be adapted depending on the required field of view. The second Intel RealSense D415 camera is mounted and calibrated with hand-eye calibration. This camera is used for robotic perception, as mentioned in 3.2.1.3. Finally, an Intel Realsense D435 camera is mounted facing the user frontally and used for teleoperation of the robot TCP *via* hand movements (gesture control). An ArUco marker is used as a reference for the finger-based teaching approach mentioned in 3.2.1. A NLU-based speech recognition module from voice INTER connect GmbH is used (voice INTER connect GmbH (2022)). This speech recognition module supports voice recognition with different languages, voice profiles (e.g: masculine or feminine), accents. It should be taken into account that all of the mentioned hardware devices are only tentative. The setup is flexible and may be changed depending on certain use case requirements. Different robotic platforms, cameras, and speech recognition systems should be compatible with the proposed approach, as the system is modular and uses standard interfaces.

### 4.2 Operation modes

Three operation modes have been implemented based on the proposed architecture mentioned in 3.1. These operation modes are:

1. Teaching mode
2. Teleoperation mode
3. Playback mode

In the teaching mode, the robotic program can be created by using index finger’s gesture and voice recognition system. Teleoperation mode supports remote control of the robot by utilizing hand gesture and voice recognition system. The playback mode is used to replay the programmed robot path in the teaching mode. A graphical user interface is utilized to give feedback and instructions to the user, manually check system status and set system parameters.

#### 4.2.1 Teaching mode

**FIGURE 6:**
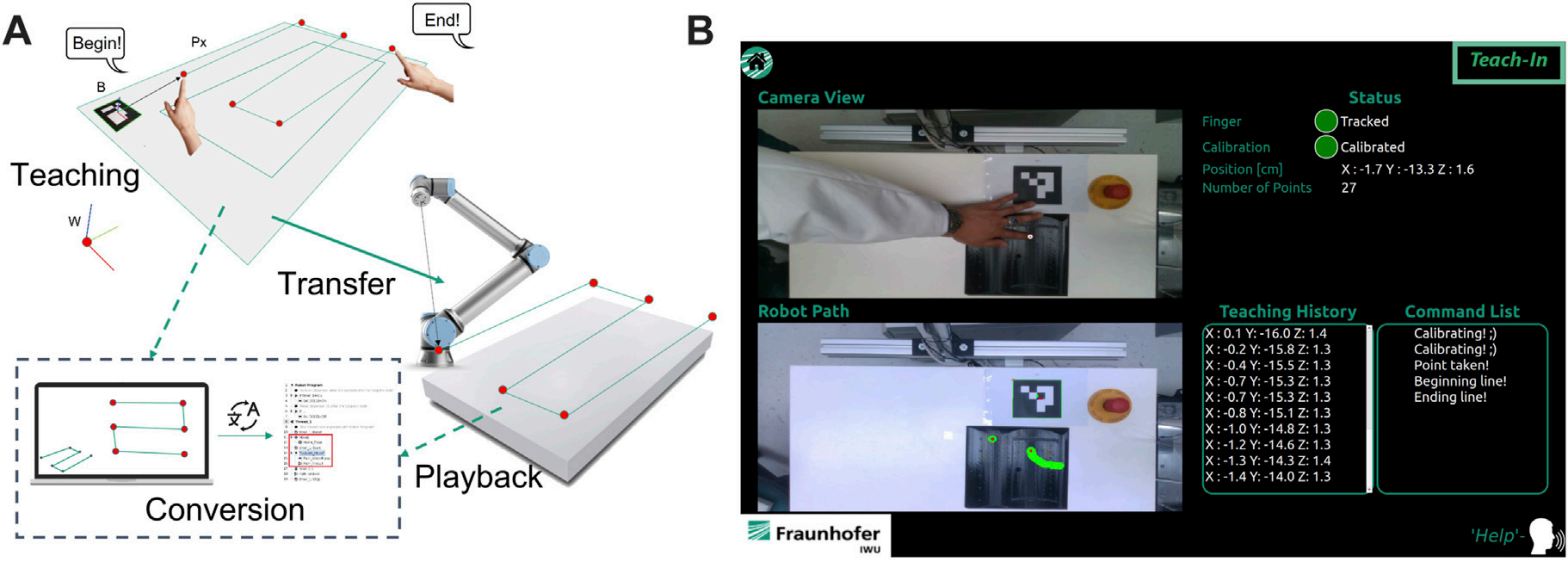
**(A)** illustration of the teaching process in teaching mode, **(B)** user interface of teaching mode.

In teaching mode, index finger’s gesture is utilized to create a robot path. By using the proposed algorithm in 3.2.1.3, the pose of the pointing finger in the teaching process can be estimated and recorded after the command is given. The voice recognition system is linked to the finite state machine and will trigger a defined action, if the command matches with the database in the context manager. As an example, command “take” triggers the state machine to extract the current pose of the finger as single robot path point. In Figure 6A, the teaching pipeline for the teaching mode and the implemented user interface are illustrated. After the teaching process is finished, the captured points are ready to be converted into robot paths in playback mode.

The implemented user interface provides real-time camera view for the teaching process and information regarding the created robot path. Additionally, information such as number of taken points, actual state of state machine, tracking status, calibration status and actual position of pointing finger are also provided *via* graphical user interface. Before the user interface of the selected operation mode is initialized, a tutorial video is played to explain to the user how the system works. If the user requires further assistance to use the system, a command list is accessible by giving a voice command “help.” The implemented actions and voice commands for the teaching mode are:

• **Calibrate**: triggers the calibration process of the individual finger profile. It should be taken into account that finger profile of each user is varied. To compensate the ground truth effect, a calibration is performed in a defined time interval. Hence, the finger profile is registered as the offset in the pose estimation mentioned in 3.2.1.2.
• **Get**: triggers the extraction of the actual position of the index finger as a single point into the currently recorded robot path.
• **Begin**: initializes the extraction of a spline. The spline is created by demonstrating the path *via* the index finger’s movement. Finger coordinates in each cycle time are extracted into the robot path until the stop command (**End**) is given. The recording process will be interrupted when the finger tracking is lost, and the taken points will not be registered in the robot path.
• **End**: ends the recording process of the spline.
• **Delete**: triggers the system to delete the latest taken object from the robot path. In this context, the object can be a single point or a spline.
• **Help**: triggers the system to show a command list for all available commands and their definitions.
• **Home**: stops the teaching mode and initialize the main menu (idle).

#### 4.2.2 Teleoperation mode

**FIGURE 7:**
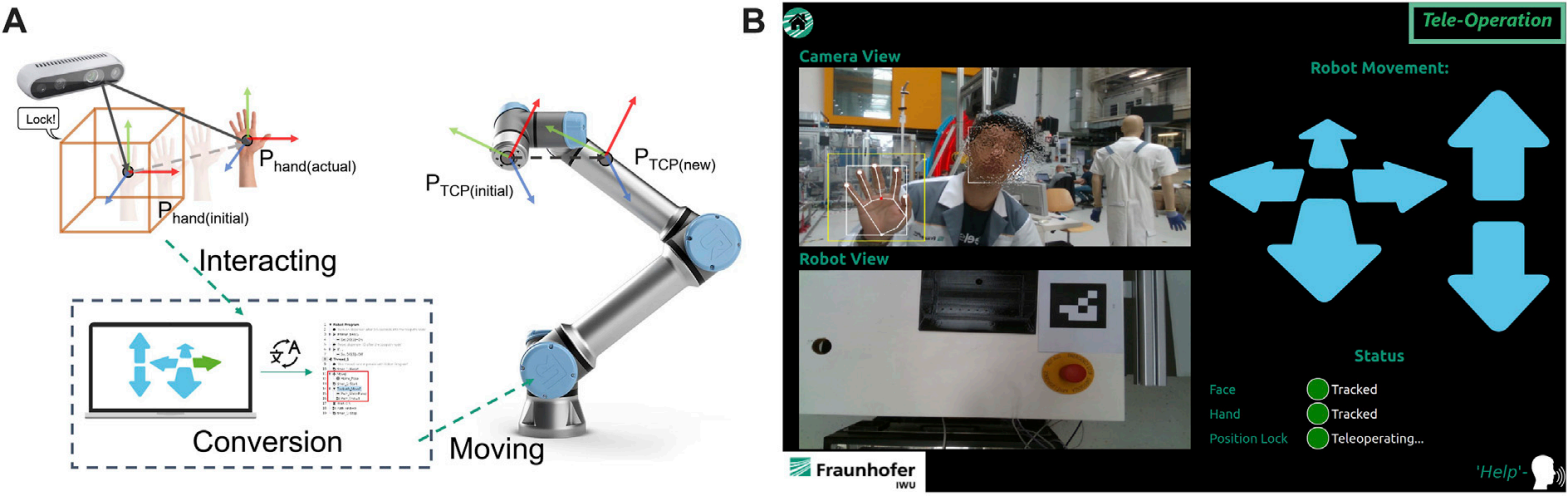
**(A)** illustration of the interaction process in teleoperation mode, **(B)** user interface of teaching mode.

In the teleoperation mode, the user can teleoperate the robot using hand gestures. A voice command is used to start the interaction. After initialization the initial position of the hand is registered and a bounding box is displayed on the feedback interface, representing the initial position of the user’s hand. The relative position of the hand to the initial position (bounding box) is calculated and used to manipulate the robot TCP in 3D. Additionally, manipulation of the robot arm’s single or multiple axes is possible. Figure 7 shows the interaction workflow, and graphical user interface for teleoperation mode.

The registered commands for teleoperation mode are:

• **Lock**: triggers the system to register the initial position of the user’s hand for the TCP manipulation.
• **Help**: trigger the system to change the manipulation mode of the system from translation into rotation or vice versa.
• **Help**: triggers the system to show a command list for all available commands and their definitions.
• **Home**: stops the teaching mode and initialize the main menu (idle).

#### 4.2.3 Playback mode

In the playback mode, the robot path created *via* teaching mode can be converted into robot specific language and further parameterized. After the “play” command, the robot path is automatically converted into a specific robotic programming language and deployed to the robot controller. Parameters such as robot speed, interpolation parameters and blending parameters are configurable *via* voice command.

### 4.3 System diagram and finite state machine (FSM)

**FIGURE 8:**
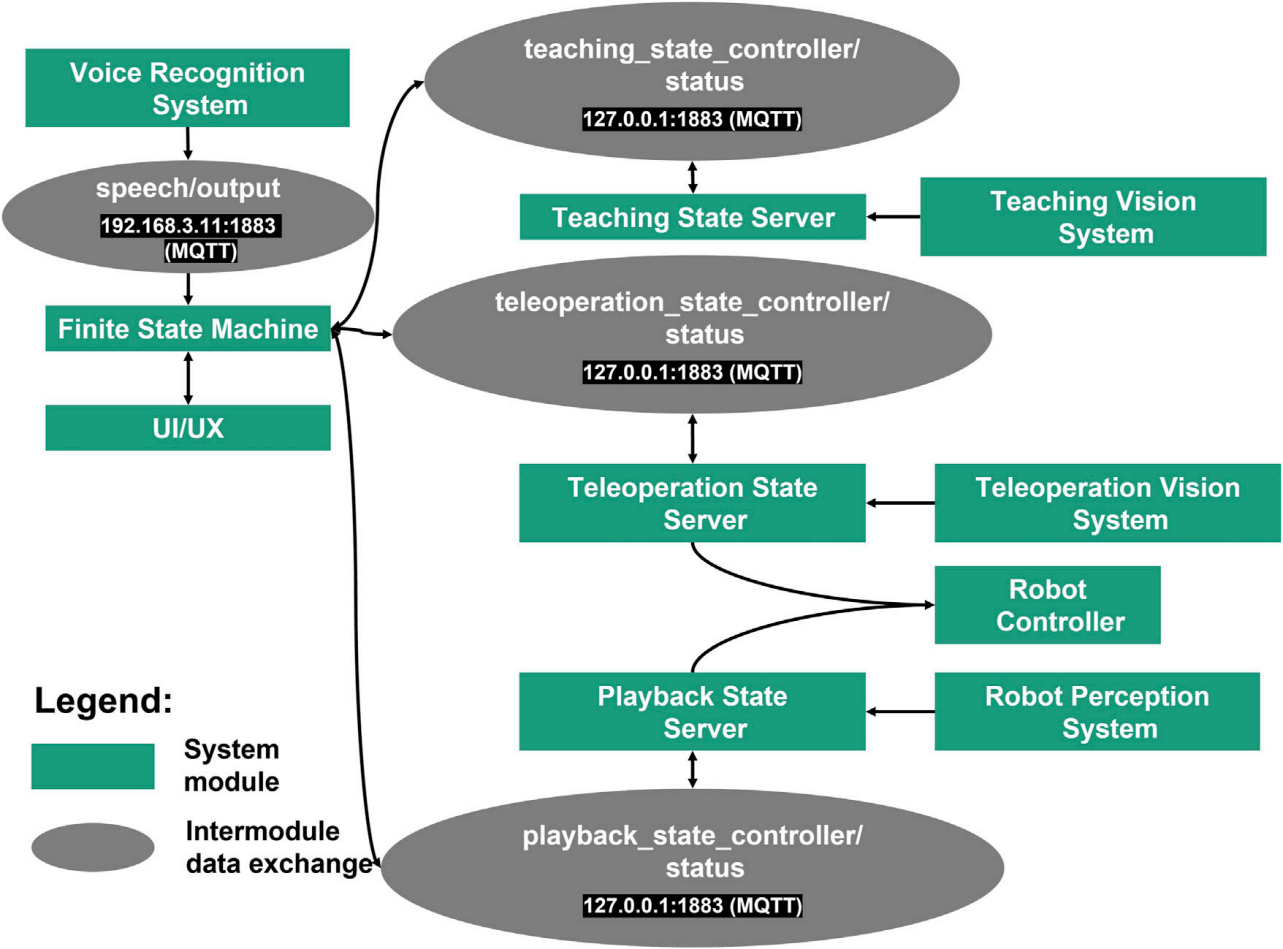
Implemented system diagram.

The implemented system diagram is shown in Figure 8. To achieve system modularity, the operation modes and other functionalities are encapsulated as system modules. For intercommunication between each module Message Queuing Telemetry Transport (MQTT) protocol was used to guarantee robust information exchange (Standard (2014)).

**FIGURE 9:**
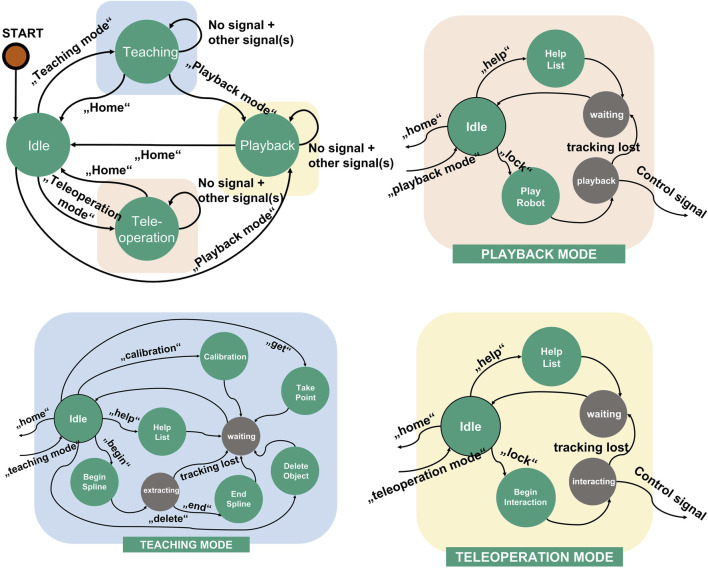
Finite state machine of the proposed system and its sub-finite state machines for each operation mode.

A finite state machine allows complexity reduction in the deployment of the robotic system (Balogh and Obdržálek (2018)). Therefore, a finite state machine is used to integrate and control all modules. Figure 9 shows the finite state machine of the whole system and its sub-finite state machines. Each operation mode mentioned in 4.2 is encapsulated as system module containing a subordinate finite state machine. Each module contains sub-modules that support the functionality of the system module for each operation mode, e.g. for the vision system and robot control. The teaching state server, teleoperation state server and playback state server receive a bypass information from the finite state machine when the respected operation mode is triggered. The bypass information is used as transition signal for each sub-finite state machine in each operation mode. In teleoperation mode and playback mode, a control system signal is sent to the robot immediately after it is triggered by interactions. The finite state machine shown in Figure 9 represents the implementation of the proposed system in this work. In the implementation, three operation modes are implemented by utilizing hand gestures, finger gestures and speeches as interaction modalities. Since the system is modular, each extension or customization in the system architecture will affect the finite state machine. In case of extension with additional systems and functionalities, the states and signals must be extended.

## 5 Results

### 5.1 Accuracy and precision assessment

**FIGURE 10:**
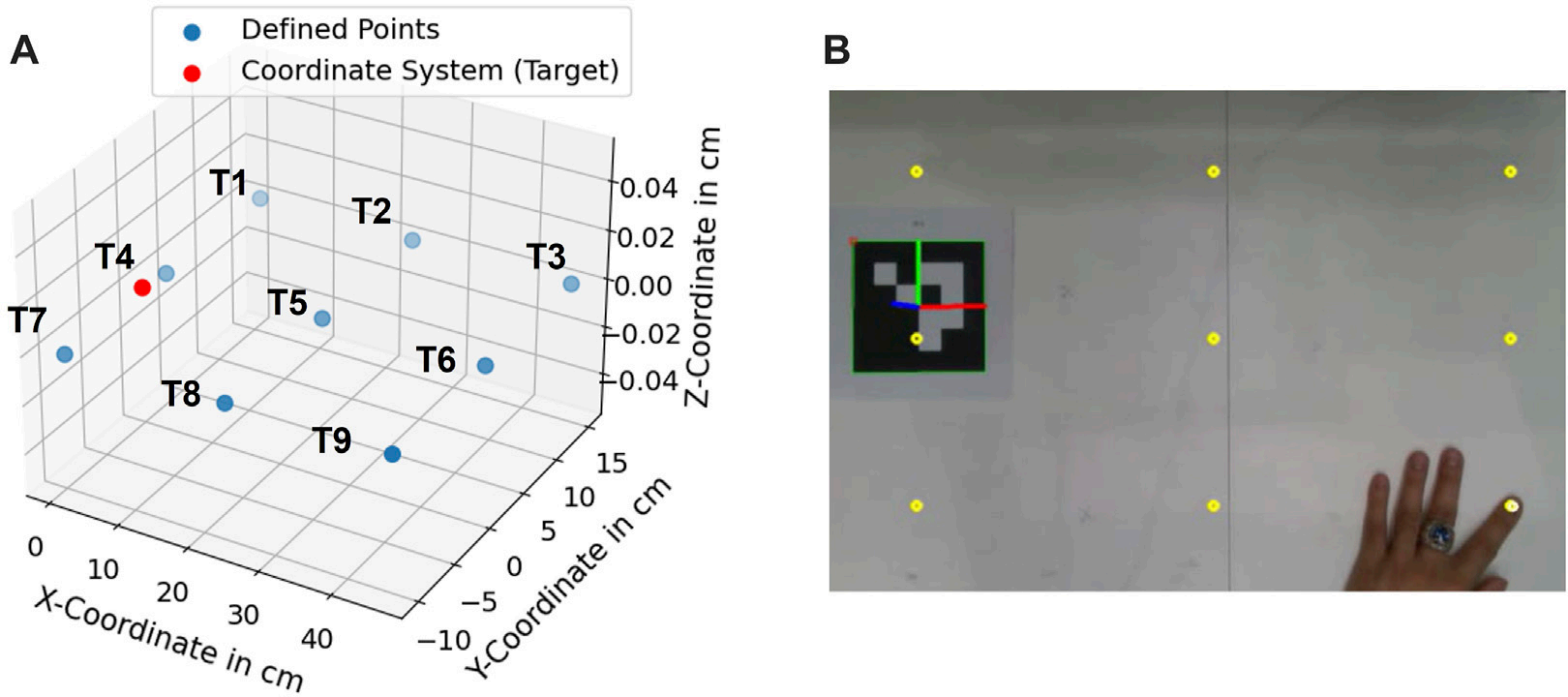
**(A)** defined coordinates (*T*1, …, *T*9), **(B)** pointing experiment at defined target points.

In order to assess the accuracy of the proposed hand- and finger tracking algorithm in 3.2.1.3, a pointing task was defined as in Figure 10. In this task, nine target coodinates (*T*1, …, *T*9) were predefined and should be pointed as accurately as possible 10 times at each point. Afterwards, the average position deviation in cm $\bar{P_{i}}$ was calculated by using euclidean norm for position deviations for each axis (Δ*x*, Δ*y*, Δ*z*) as shown in Eq. 9.

$$\bar{P_{i}}=\sqrt{{\Delta x}^{2}+{\Delta y}^{2}+{\Delta z}^{2}}$$ (9)

**FIGURE 11:**
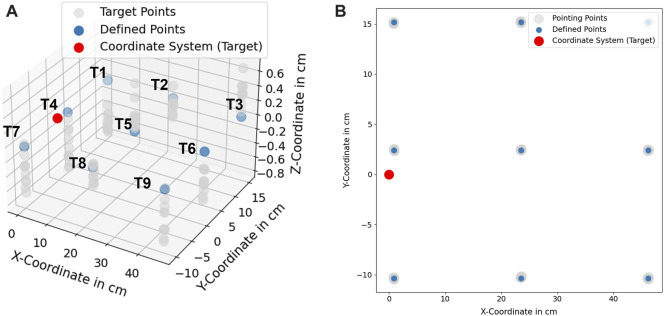
**(A)** Scaled position deviation at defined coordinates (T1, ..., T9), **(B)** 2D-View of scaled position deviation at defined coordinates.

The measurement was performed with camera height at 65 *cm*. The light intensity measured in the environment was 580 *Lux* at 1,5 *m* above the floor and the temperature was at 21*°C*. In Figure 11, the measured coordinates are compared with the defined coordinates in 3D and 2D. As a result, the spatial information of the pointed coordinates at the *z*-axis is more inaccurate in comparison to the information at the x- and *y*-axis. The inaccuracy is caused due to the noise from the depth information obtained from the camera. From the technical specification of Intel RealSense D415, the depth accuracy from the camera is 2% < 2*m* (Intel (2015)).

A recent study for the performance of Intel RealSense D415 showed that for the short distance 500–1000 *mm*, the camera delivers up to 30 *mm* deviation in depth estimation (Servi et al. (2021)). From the obtained results, it can be concluded that the accuracy of the proposed method achieves 3.71 ± 2.07 *mm*. The statistical analysis of each target point is shown in Table 1.

**TABLE 1:** Measurement uncertainty for accuracy measurement at each target point.

Point	Euclidean deviation $\bar{P_{i}}$ [*mm*]	Standard deviation $\sigma_{P_{i}}$ [*mm*]
T1	6.43	0.96
T2	1.99	2.25
T3	3.63	1.77
T4	2.52	1.45
T5	2.44	1.72
T6	1.89	0.61
T7	3.49	1.57
T8	4.44	1.41
T9	3.88	2.15
Σ	3.71	2.07

The resulting deviations in the system can be caused by several factors. A human can not point a target point accurately with its finger, caused by the anatomy of the human finger. This uncertainty can be varied in the range of *mm* and *cm* depending on the human hand-eye coordination skill or the dexterity of the user. A further observation was performed to assess the systematical deviations (precision) from the proposed algorithm in 3.2.1.3. A new assessment task was formulated. In this task, nine target coordinates T1…T9 were used. A finger was pointing to these points, and the finger was maintained to be static while the finger’s position was being recorded. In Figure 14, standard deviations of the measured points at the x- and *y*-axis are shown with 95% confidence ellipsoid to give an overview of the system precision (*See* 95% confidence ellipsoid in 6 for reference). Standard deviation in the *z*-axis is also shown in Figure 15. Standard deviation in x-,y- and *z*-axis (*σ*_*x*_, *σ*_*y*_, *σ*_*z*_) and standard deviation of Euclidean distance in 2D (*σ*_*r*_) are represented in Table 2.The result showed that the tracking deviation at the x- and *y*-axis are smaller than the deviation at the *z*-axis. In each target point, the planar deviation is less than 1 *mm*. The deviation of the depth information is less than 2 *mm*. The deviations existed due to the inaccuracy in the intrinsic and extrinsic calibration of the camera system. The higher deviation in depth information indicated that the camera delivers inconsistent depth information. Despite the higher deviation in depth information, the result showed that the proposed image processing algorithms mentioned in 3.2.1.3 can reduce the depth inaccuracy of the camera system. In conclusion, the assessment method shows promising results of the proposed method to be deployed for robotic programming applications with relative accuracy up to 6 *mm* and the tracking system can deliver up to 2 mm precision with the defined setup in 4.1.

**TABLE 2:** Standard deviation of the tracking system precision.

Point	Number of Points	*σ*_*x*_ [*mm*]	*σ*_*y*_ [*mm*]	*σ*_*r*_ [*mm*]	*σ*_*z*_ [*mm*]
T1	99	0.48	0.44	0.65	1.09
T2	99	0.28	0.39	0.49	1.27
T3	99	0.41	0.45	0.61	1.71
T4	99	0.54	0.30	0.62	1.01
T5	99	0.54	0.31	0.62	1.17
T6	99	0.46	0.59	0.75	0.89
T7	98	0.36	0.42	0.55	1.25
T8	98	0.45	0.44	0.63	1.83
T9	99	0.42	0.20	0.46	1.68

### 5.2 Benchmarking with state-of-the-art

**FIGURE 12:**
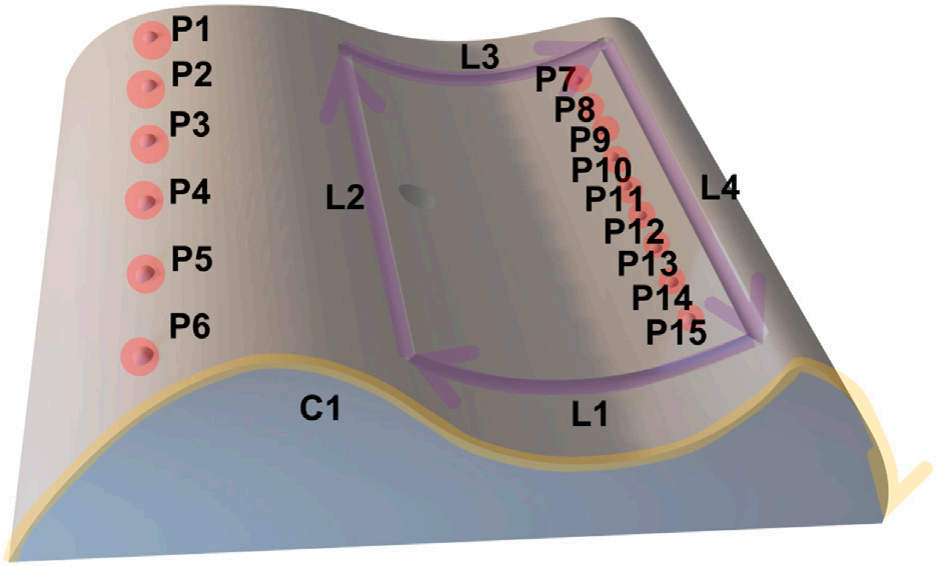
3D printed part for benchmarking assessment.

In order to show the practicability of the proposed method, a benchmarking is done by comparing the proposed system with the implemented methods from the state-of-the-art such as hand-guiding and programming by teach pendant in Universal Robots UR10 which are specified in the actual standards for industrial robot system [DIN EN ISO 10218-1 (2021); DIN EN ISO 10218-2 (2012); DIN ISO/TS 15066 (2017)]. This assessment is performed in a real-world teaching scenario for painting or gluing application in the real production. A workpiece as shown in Figure 12 was manufactured with specific features that would be used for the tasks in this assessment.

The features are 15 points (P1,…,P15), four lines with their directions (L1,…,L4) and a curve with its direction (C1). The tasks in this assessment consist of movement sequence based on these features. In total, four movement sequences with different complexity were executed by using the multimodal programming approach in this work. Each task will be repeated by using hand-guiding and online programming approach *via* teach pendant from Universal Robots UR10 controller. The number of points and execution time for each task are measured for the assessment. The tasks are described in following:

• Task 1: PTP linear movement (*P*6 → *P*5 → *P*4 → *P*3 → *P*2 → *P*1)
• Task 2: PTP zigzag movement (*P*6 → *P*15 → *P*5 → *P*13 → *P*4 → *P*11 → *P*3 → *P*9 → *P*2 → *P*7 → *P*1)
• Task 3: Movement along defined features (*L*1 → *L*2 → *L*3 → *L*4)
• Task 4: Movement along defined contour (*C*1)

The overview of the assessment result is depicted in Table 3 (detailed result in Table 7). For the assessment, the time ratio and number of point (NoP) ratio between teach pendant and hand-guiding teaching to the proposed method were calculated. The time ratio is calculated as the quotient of the mean time of the hand-guiding or teach pendant and the proposed method. For the number of point, the same normalization is performed by building quotient of number of recorded points for the programming methods from the state-of-the art and the proposed method. In the programming methods with teach pendant or hand-guiding, the user must determine how many points must be taken to extract the features of the work piece. In the proposed method, this issue does not exist because the finger’s movement along the features is extracted in the teaching process. As a result, the selected features can be extracted as coordinate points in the proposed method. Therefore, the number of points as assessment criterion is necessary to give an objective benchmark in this assessment.

**TABLE 3:** Overview for effort reduction from the proposed method (rel. Reduction (PM)) in the benchmarking assessment, TP, teach pendant; HG, hand-guiding; PM, proposed method.

Parameter	Task 1			Task 2			Task 3			Task 4		
**Method**	**TP**	**HG**	**PM**	**TP**	**HG**	**PM**	**TP**	**HG**	**PM**	**TP**	**HG**	**PM**
Mean time [s]	92.73	71.08	28.69	118.35	101.63	50.28	71.19	70.38	24.34	65.47	56.28	17.71
Time ratio	3.23x	2,47x	—	2.35x	2.02x	—	2.92x	2.89x	—	3.69x	3.18x	—
Mean NoP	6	6	6	11	11	11	6	6	88	7	7	70.33
NoP ratio	1x	1x	—	1x	1x	—	0.06x	0.06x	—	0.09x	0.09x	—
Rel. Reduction (PM)	3.23x	2.47x	—	2.35x	2.02x	—	48.67x	48.17x	—	41.00x	35.33x	—

**FIGURE 13:**
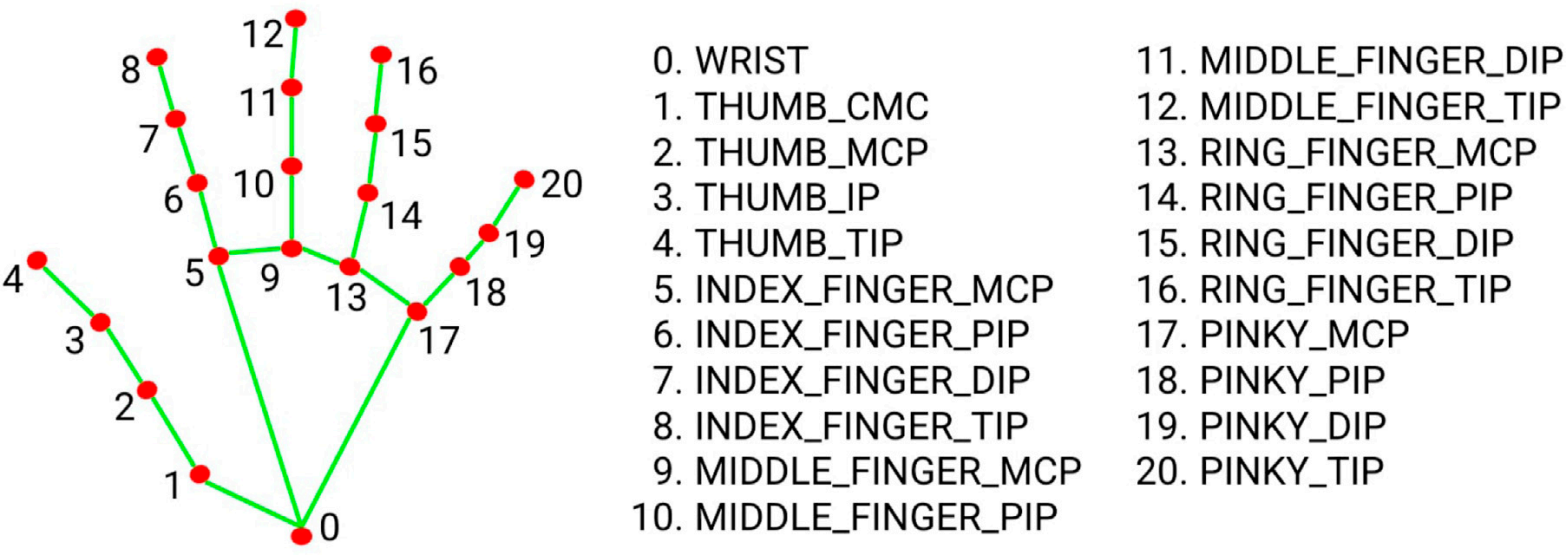
Hand model and key points of MediaPipe.

These ratios were used to calculate the relative reduction for the benchmarking using following equation:

$$Relative\;reduction=\frac{Time\;Ratio}{NoP\;Ratio}$$ (10)

For simple PTP motions in tasks 1 and 2, the proposed method showed effort reduction with 2–3× factor. In the experiments, speech commands had to be repeated several times in some cases, due to environmental noise ( >60dB ). This led to longer teaching times. A backup solution to improve performance issues caused by environmental noise is considered by utilizing alternative input interfaces such as a keyboard or other peripheries. The results from tasks 3 and 4 showed drastic improvements in the generation of complex movement profiles, such as movement along specific features. By performing the task using the programming methods from state-of-the-art, the first hindrance was to consider how many points should be extracted to build a detailed movement profile along the desired feature. The programming effort was significantly improved when more points should be extracted. In contrast, even though less programming time can be achieved by reducing the number of points, the desired movement profile will be compensated due to adequate detailed information from the taken points. This drawback effect was shown in tasks 3 and 4 using hand-guiding and teach pendant. Hereby, less than ten points were taken to generate the movement profile. Eventually, the desired movement profile could not be fulfilled due to sufficient information on the desired feature. In comparison to the methods from state-of-the-art, the proposed method showed incisive results with 40–50× effort reduction for complex tasks such as tasks 3 and 4. In the proposed method, the desired feature can be extracted as a robot movement profile by tracking the finger movement on the corresponding feature directly. The proposed multimodal no-code programming approach showed the potential to drastically reduce the teaching time and effort for robotic programs compared to the state-of-the-art.

**FIGURE 14:**
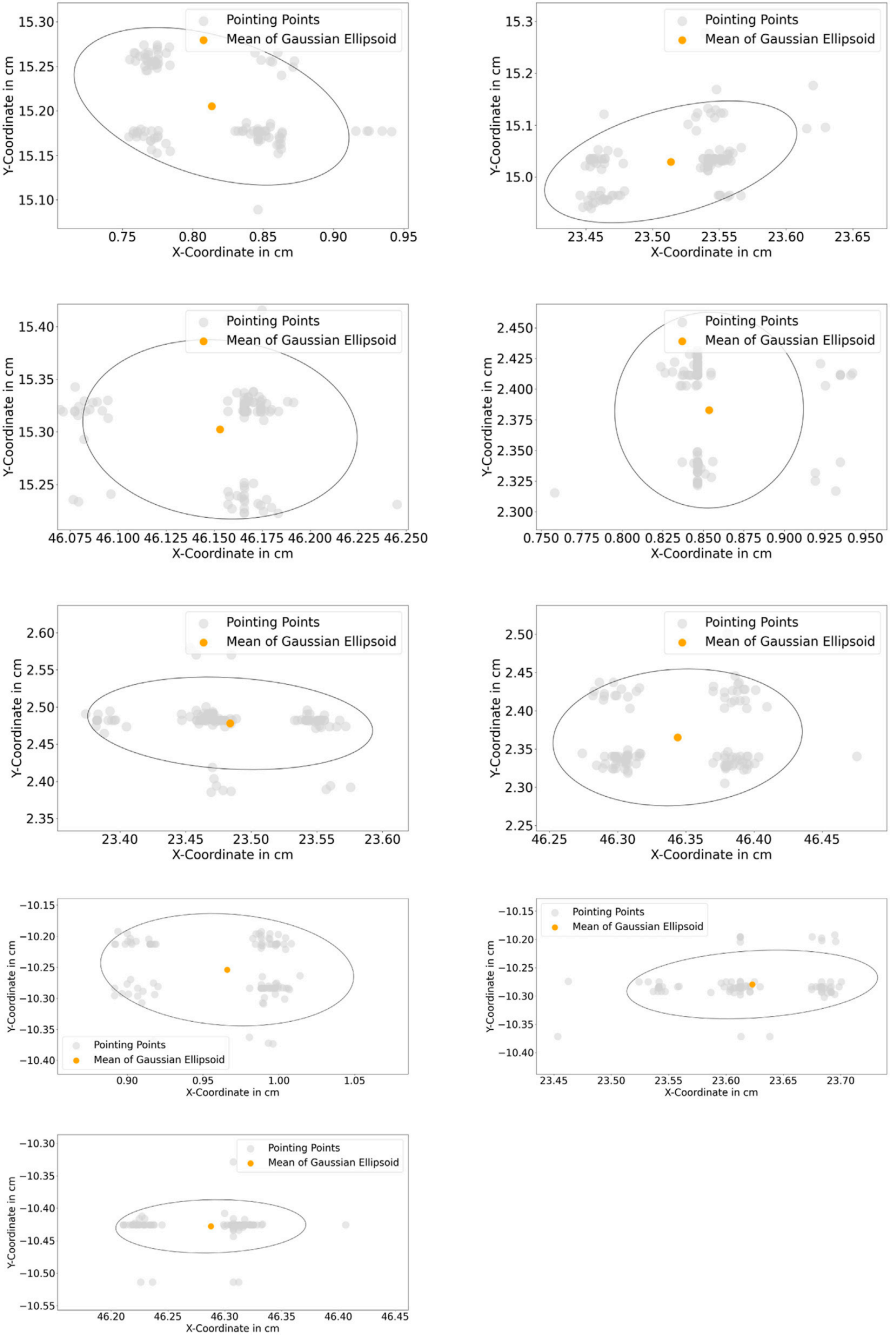
(Top left to bottom right): 95% gaussian ellipsoid for measurement of standard deviation of static points from T1…T9.

## 6 Discussions and conclusions

In many cases, the intuitive teaching methods from state-of-the-art are not ready to be implemented directly in an industrial environment. The proposed programming approaches from the state-of-the-art are mostly task-oriented and can be performed only to create a robot routine for a specific process. The system setups are fixed with strictly defined sensors, and there is no room for customization. Even though the proposed systems prioritize ease of use and consider intuitive interactions in the teaching process, many works are not implementable in industrial environments due to non-practicable methodologies and complex system configurations. These hurdles are antitheses to the concepts of HRC, which enables robotic systems to be agile, reconfigurable and adaptable when changes in production occur. This work proposes a novel approach to intuitive programming by utilizing multimodal interactions such as speech and gestures. The proposed programming approach introduces a generic teaching solution for HRC applications in agile production by utilizing low-cost sensors. The novel approach allows the user to (re-)configure the robot program in the scenario where major or minor changes occur in production.

**FIGURE 15:**
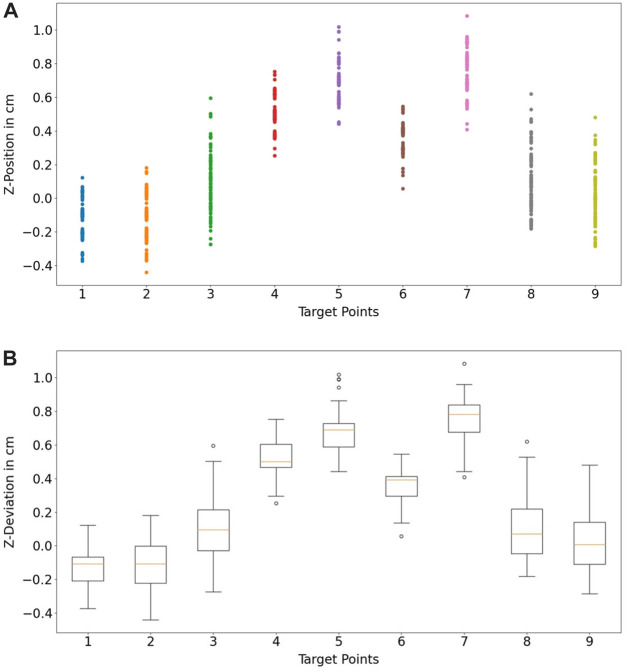
**(A)** depth information of the static point measurement at each target point, **(B)** standard deviation each static point in box plots.

Compared to state-of-the-art robotic programs, such as teach pendant and hand-guiding, the novel method proposes in this work showed that the programming effort for complex tasks can be reduced by 40–50 times. It also enables non-robotic experts to reconfigure and create robotic programs in a short time using multimodal interaction. With the approach robot paths can be taught by demonstration of finger gestures with 6 *mm* accuracy. The proposed computer vision algorithm for hand- and finger-gesture estimation has thus shown its capability to achieve a precision up to 2 *mm* in the observed environment. In comparison to alternative no-code robotic programming approaches in the state of the art, the results with the low-cost hardware in the current setup (*see* 4.1) show great potential for no-code robotic programming. The analysis of the extracted orientation in the hand- and finger-gesture estimation will be addressed in the future work by comparing a single camera setup and multi-camera setup. This comparison will give a clear overview for the singularity issues in the extraction of finger orientation. The proposed system provides a modular and expandable system setup, utilizing low-cost hardware, in contrast to many state-of-the-art reference papers. Hence, the algorithms can be applied, extended and modified to fit different applications and scenarios by using different sensor technologies, robot systems and tools for example: the speech recognition system can be substituted by other low-cost input modalities (e.g: keyboard, button), the current low-cost cameras can be upgraded with high-end industrial cameras, the current robot system can be replaced by different cobots or traditional industrial robots, and linear axes can be integrated in the system.

In a robotic-applied industrial process, process parameters and requirements should be controlled to guarantee the quality of the end product. The robotic experts should not only be proficient in creating robotic programs, but they should also integrate the process parameter in the manufacturing process to meet the aimed quality of the end product. Even though robotic programming methods from state-of-the-art have simplified robotic programming for experts, The harmonization of the process parameter is still a big topic to research in the robotic research community. Most of the introduced approaches from the state-of-the-art are focusing only in developing a task oriented solutions for a specific application (e.g., Pick-and-Place and assembly). In contrast to them, the proposed method in this work offers a new perspective for a generic solution in intuitive robot programming by addressing modularity, agility and flexibility in the system setup. As a result, integration or replacement with different systems (e.g., sensors, robots) are possible. The modularity allows the programming approach to be combined with another algorithm (skill sets) to resolve an issue for robot program with specific applications. In Table 4, robotics-based industrial applications from different works in recent years are shown with their tolerance ranges. By comparing the result from the accuracy assessment of the novel approach with the given tolerances, it can be concluded that the proposed method has enormous potential to be implemented in various applications where medium tolerances in 10 *μm* ≤ *x* ≤ 10 *mm* and coarse tolerances in *x* > 10 *mm* are required. On the other side, the 6 *mm* accuracy of the proposed method would not satisfy the requirement for processes with fine tolerance in *x* < 10 *μm*. Even though the current work was focused on the proposed method of teaching the robotic path based on hand-finger-gesture and voice. The vision and speech modality used in this work allows further development of intuitive robotic skill sets for the applied industrial processes in future works. These skill sets will allow the user to parameterize their process parameters and execute the process by applying process-specific control strategies as shown in Table 4. An example of a welding application will be explained in the following to depict the potential improvement of the system’s inaccuracy by developing a welding skill set. The user would draw a welding path on the welding joint using his/her finger. The user triggers the skill set by saying “welding mode on.” The finite state machine may trigger the activation of the vision-based control system to follow the weld, e.g. by using the methods mentioned in Table 4. This weld tracking algorithm will be used as a reference to control and compensate for the inaccuracy from the teaching phase. Another example represents an intuitive skill set for polishing that would allow automatic generation of process paths for basic geometries based on single user-defined points or features on the work piece *via* finger tracking. Trajectories with higher complexity may be taught to the robotic system by combining finger gestures and online impedance control of the robot manipulator. Specific parameters, e.g. amount of applied force for impedance control, may be figured by the user *via* voice commands. The combination of the multimodal programming method in this paper with intuitive skill sets will accelerate the deployment and reconfiguration of robotic systems in industrial context. In the future work, the implementation of intuitive skill sets for the proposed method will be addressed and assessed in an industrial use-case.

**TABLE 4:** Potential industrial applications for the proposed method with the estimated process tolerances and possible control strategies for development of skill based technology modules.

**Applications**	**Tolerance**	**Skill based control strategy**
Handing-over	⊘, ⊗	Bdiwi et al. (2013a,c)
Manipulation	⊙, ⊘, ⊗	Jokesch et al. (2014)
Painting	⊘, ⊗	Zhang et al. (2020a); Tadic et al. (2021)
Peg-in-hole	⊘	Bdiwi et al. (2015); Haugaard et al. (2020)
Polishing	⊘, ⊗	Tian et al. (2016); Kakinuma et al. (2022); Zhou et al. (2021)
Welding	⊙, ⊘	Yang et al. (2018b); Lei et al. (2021)

⊙ - fine (*x* < 10 *μm*), ⊘ - medium (10 *μm* ≤ *x* ≤ 10 *mm*), ⊗ - coarse (*x* > 10 *mm*).

**TABLE 5:** System requirement for the vision system.

Requirement of vision system **Parameter**	**Min. value**	**Description**
Camera RGB Resolution	1280 × 720 (HD)	Since the hand-tracking system algorithm works with RGB images as input the higher resolution offers better performance in tracking
Camera depth resolution	640 × 480	The higher depth resolution delivers higher details of in-depth information, otherwise, it costs a longer computation time to obtain this information
Camera RGB field of view	60° × 40°	The field of view (FoV) describes the possible detection area that the camera image could deliver. The higher FoV offers a wider detection area for the system
Camera depth field of view	50° × 30°	Since RGB and depth FoV are not exactly the same, a synchronization function to map both information should be performed to get more detail mapping of both information
Camera RGB/deph frame rate	30 FPS	A higher frame rate would cause less latency. In other words, a higher frame rate would improve the system response in image processing
Camera min depth information	x > 0.2 m	The actual stereo camera system has minimum depth information that can be obtained. The nearer the minimum available depth information the more accurate the calculation for the spatial information of the hand and finger tracking
Hand tracking model	All phalanx on handshould be available	The hand tracking system should deliver as many landmarks (phalanxes). Since the proposed approach with the deictic gesture of a pointing finger, the landmarks in the pointing finger should be able to be identified
Hand tracking stability	Stabile in all light conditions	The hand tracking system should work with RGB images in different lighting conditions
Hand tracking distance	0–1.5 m	The tracking system should work at a farther distance to compensate for the deficiency due to the minimum distance from the depth information of the stereo camera
Hand tracking frame rate	30 FPS	The higher the frame rate the hand tracking system could deliver, the more fluent the interaction between end-user and system could occur

The camera-based vision system showed great potential for implementing the LfD strategy for robotic applications compared to other technology such as VR-, AR- or XR-based motion capture, used in state-of-the-art. However, the camera system still has its characteristic limitations in certain aspects. Various vision-based algorithms have pushed the vision system’s limits and can compensate for many drawbacks of camera systems. In future works, an improvement in the methodology of the vision system can be addressed by applying recent algorithms from the state-of-the-art, such as:

• Positional and rotational accuracy improvement of the system → implementation of multi-camera system (Lippiello et al. (2005); Hoang (2020)), usage of camera with different technology (Langmann et al. (2012); Lourenço and Araujo (2021))
• Translation and rotation of the component after teaching → implementation of 6D object pose algorithm (Xiang et al. (2017); Sun et al. (2022))
• Component is bigger than field of view of the camera → usage of additional axes on a workpiece fixture or camera (translating the object with respect to the camera or *vice versa*) and implementation of image stitching or photogrammetry algorithm (Li et al. (2017); Ding et al. (2019))

**TABLE 6:** System requirement for the voice recognition system.

Requirement of the voice recognition system		
**Parameter**	**Value**	**Description**
Recognition type	Offline	Since the recognition sytem is used in an industrial context, an offline voice recognition system is demandable to maintain data security
Dialogue design	Conformed based on ISO/IEC 30122	The dialogue should be designed as easily as possible as mentioned in ISO/IEC 30122
Dialogue extraction	Text-to-speech for every uttered words	The system should be able to extract single word in a sentece uttered by the end-user

In conclusion, this work contributes a novel approach to multimodal robotic programming by utilizing hand-finger-gesture recognition and speech recognition which can be implemented in different industrial applications and robotic systems. The proposed method is suitable for use without or with adequate experts in robotic programming. The *bona fide* evaluation results showed the system’s potential to replace actual state-of-the-art methods. The opportunities for future developments of the system depict that the system can be a game changer in industrial robotic programming. This proposed programming method will accelerate the deployment of robotic systems in industrial use-case and affect how robotic systems are programmed in the industry for serial production or even batch size 1.

**TABLE 7:** Benchmarking result with comparison at programming time and number of taken points (NoP), TP, Teach Pendant; HG, hand-guiding; PM, Proposed Method.

**Task**	**Method**	**Time 1 [s]**	**Time 2 [s]**	**Time 3 [s]**	**Mean time [s]**	**NoP 1**	**NoP 2**	**NoP 3**	**Mean NoP**
Task 1	TP	90.05	97.06	89.12	92.73	6	6	6	6
									
									
	HG	75.46	70.30	77.49	71.08	6	6	6	6
									
									
	PM	28.28	28.68	29.12	28.69	6	6	6	6
Task 2	TP	122.61	110.18	102.25	101.63	11	11	11	11
									
									
	HG	101.32	101.33	102.25	118.35	11	11	11	11
									
									
	PM	48.56	55.82	46.46	50.28	11	11	11	11
Task 3	TP	78.73	77.58	75.25	77.19	6	6	6	6
									
									
	HG	69.56	74.84	66.74	70.38	6	6	6	6
									
									
	PM	24,95	26,34	21.73	24.34	85	97	82	88
Task 4	TP	67.85	58.30	70.25	65.47	7	7	7	7
									
									
	HG	58.49	55.81	54.53	56.28	7	7	7	7
									
									
	PM	21.19	15.93	16.02	17.71	87	59	65	70.33

## Data Availability

The original contributions presented in the study are included in the article/Supplementary Material, further inquiries can be directed to the corresponding author.

## References

[B1] Ajaykumar G. Stiber M. Huang C.-M. (2021). Designing user-centric programming aids for kinesthetic teaching of collaborative robots. Robotics Aut. Syst. 145, 103845. 10.1016/j.robot.2021.103845

[B2] Akkaladevi S. C. Pichler A. Plasch M. Ikeda M. Hofmann M. (2019). Skill-based programming of complex robotic assembly tasks for industrial application. Elektrotech. Inftech. 136, 326–333. 10.1007/s00502-019-00741-4

[B3] Akkaladevi S. C. Plasch M. Chitturi N. C. Hofmann M. Pichler A. (2020). Programming by interactive demonstration for a human robot collaborative assembly. Procedia Manuf. 51, 148–155. 10.1016/j.promfg.2020.10.022

[B4] Andreff N. Horaud R. Espiau B. (1999). “On-line hand-eye calibration,” in Second International Conference on 3-D Digital Imaging and Modeling (No.PR00062), (Ottawa, Canada), 430–436. 10.1109/IM.1999.805374

[B5] Angelidis A. Vosniakos G.-C. (2014). Prediction and compensation of relative position error along industrial robot end-effector paths. Int. Suchy’ J. Precis. Eng. Manuf. 15, 63–73. 10.1007/s12541-013-0306-5

[B6] Argall B. D. Chernova S. Veloso M. Browning B. (2009). A survey of robot learning from demonstration. Robotics Aut. Syst. 57, 469–483. 10.1016/j.robot.2008.10.024

[B7] Balogh R. Obdržálek D. (2018). “Using finite state machines in introductory robotics,” in International Conference on Robotics and Education RiE 2017 (Sofia, Bulgaria: Springer), 85–91.

[B8] Bdiwi M. Hou S. Winkler L. Ihlenfeldt S. (2021). “Empirical study for measuring the mental states of humans during the interaction with heavy-duty industrial robots,” in 2021 IEEE Conference on Cognitive and Computational Aspects of Situation Management (CogSIMA) (Talinn, Estonia: IEEE), 150–155.

[B9] Bdiwi M. Kolker A. Suchý J. Winkler A. (2013a). “Automated assistance robot system for transferring model-free objects from/to human hand using vision/force control,” in Social Robotics (Cham: Springer International Publishing), 40–53.

[B10] Bdiwi M. Kolker A. Suchý J. Winkler A. (2013b). “Segmentation of model-free objects carried by human hand: Intended for human-robot interaction applications,” in 2013 16th International Conference on Advanced Robotics (ICAR) (Montevideo, Uruguay: IEEE), 1–6.

[B11] Bdiwi M. Suchỳ J. Jockesch M. Winkler A. (2015). “Improved peg-in-hole (5-pin plug) task: Intended for charging electric vehicles by robot system automatically,” in 2015 IEEE 12th International Multi-Conference on Systems, Signals & Devices (SSD15) (Mahdia, TN: IEEE), 1–5.

[B12] Bdiwi M. Suchỳ J. Winkler A. (2013c). “Handing-over model-free objects to human hand with the help of vision/force robot control,” in 10th International Multi-Conferences on Systems, Signals & Devices 2013 (SSD13) (Hammamet, TN: IEEE), 1–6.

[B13] Beck J. Neb A. Barbu K. (2021). Towards a cad-based automated robot offline-programming approach for disassembly. Procedia CIRP 104, 1280–1285. 10.1016/j.procir.2021.11.215

[B14] Billard A. Calinon S. Dillmann R. Schaal S. (2008). “Robot programming by demonstration,” in Springer handbook of robotics.

[B15] Blankemeyer S. Wiemann R. Posniak L. Pregizer C. Raatz A. (2018). Intuitive robot programming using augmented reality. Procedia CIRP 76, 155–160. 10.1016/j.procir.2018.02.028

[B16] Bolano G. Roennau A. Dillmann R. Groz A. (2020). “Virtual reality for offline programming of robotic applications with online teaching methods,” in 2020 17th International Conference on UbiquitousRobots (UR) (Kyoto, Japan: IEEE), 625–630.

[B17] Cho J.-M. Park S.-Y. Chien S.-I. (2020). Hole-filling of realsense depth images using a color edge map. IEEE Access 8, 53901–53914. 10.1109/ACCESS.2020.2981378

[B18] Chryssolouris G. Georgoulias K. Michalos G. (2012). “Production systems flexibility: Theory and practice,” in IFAC Proceedings 14th IFAC Symposium on Information Control Problems in Manufacturing, 15–21. 10.3182/20120523-3-RO-2023.00442

[B19] Daniilidis K. (1999). Hand-eye calibration using dual quaternions. Int. J. Robotics Res. 18, 286–298. 10.1177/02783649922066213

[B20] Dietz T. Schneider U. Barho M. Oberer-Treitz S. Drust M. Hollmann R. (2012). “Programming system for efficient use of industrial robots for deburring in sme environments,” in ROBOTIK 2012; 7th German Conference on Robotics.

[B21] DIN EN ISO 10218-1 (2012). *DIN EN ISO 10218-1: Robotik sicherheitsanforderungen - teil 1: Industrieroboter* norm. Geneva, CH: International Organization for Standardization.

[B22] DIN EN ISO 10218-2 (2021). Industrieroboter - sicherheitsanforderungen - Teil 2: Robotersysteme und Integration. Geneva, CH: Norm, International Organization for Standardization.

[B23] DIN ISO/TS 15066 (2017). Din ISO/TS 15066: Roboter und Robotikgeräte - kollaborierende Roboter. Norm. Geneva, CH: International Organization for Standardization.

[B24] Ding C. Liu H. Li H. (2019). Stitching of depth and color images from multiple rgb-d sensors for extended field of view. Int. J. Adv. Robotic Syst. 16, 172988141985166. 10.1177/1729881419851665

[B25] Du G. Zhang P. (2014). A markerless human–robot interface using particle filter and kalman filter for dual robots. IEEE Trans. Ind. Electron. 62, 2257–2264. 10.1109/tie.2014.2362095

[B26] El Zaatari S. Marei M. Li W. Usman Z. (2019). Cobot programming for collaborative industrial tasks: An overview. Robotics Aut. Syst. 116, 162–180. 10.1016/j.robot.2019.03.003

[B27] Elliott S. Xu Z. Cakmak M. (2017). “Learning generalizable surface cleaning actions from demonstration,” in 2017 26th IEEE International Symposium on Robot and Human Interactive Communication (RO-MAN (IEEE), 993.

[B28] Funes-Lora M. Vega-Alvarado E. Rivera-Blas R. Calva-Yáñez M. Sepúlveda-Cervantes G. (2021). Novel surface optimization for trajectory reconstruction in industrial robot tasks. Int. J. Adv. Robotic Syst., 18. Publisher Copyright: ⓒ The Author(s). 10.1177/17298814211064767

[B29] Garrido-Jurado S. Muñoz-Salinas R. Madrid-Cuevas F. J. Marín-Jiménez M. J. (2014). Automatic generation and detection of highly reliable fiducial markers under occlusion. Pattern Recognit. 47, 2280–2292. 10.1016/j.patcog.2014.01.005

[B30] Gastal E. S. L. Oliveira M. M. (2011). Domain transform for edge-aware image and video processing. ACM Trans. Graph. 30, 1–12. 10.1145/2010324.1964964

[B31] Hägele M. Nilsson K. Pires J. N. Bischoff R. (2016). Industrial robotics. Cham: Springer International Publishing. –1422. 10.1007/978-3-319-32552-1_54

[B32] Haugaard R. L. Langaa J. Sloth C. Buch A. G. (2020). Fast robust peg-in-hole insertion with continuous visual servoing. *arXiv preprint arXiv:2011.06399* .

[B33] Hoang V. T. (2020). Hgm-4: A new multi-cameras dataset for hand gesture recognition. Data Brief 30, 105676. 10.1016/j.dib.2020.105676 32435681 PMC7229479

[B34] Horaud R. Dornaika F. (1995). Hand-eye calibration. Int. J. Rob. Res. 14, 195–210. 10.1177/027836499501400301

[B35] Hornung L. Wurll C. (2022). “Human-robot collaboration: a survey on the state of the art focusing on risk assessment,” in Proceedings of Robotix-Academy Conference for Industrial Robotics (RACIR) 2021, Duren, Germany: Shaker Verlag, 10–17.

[B36] Huang W. Ren P. Wang J. Qi Q. Sun H. (2020). Awr: Adaptive weighting regression for 3d hand pose estimation.” in AAAI Conference on Artificial Intelligence (AAAI). IEEE.

[B37] Infante M. L. Kyrki V. (2011). “Usability of force-based controllers in physical human-robot interaction,” in 2011 6th ACM/IEEE International Conference on Human-Robot Interaction (HRI) (Lausanne, Switzerland: IEEE), 355–362.

[B38] Intel (2015). Intel RealSense D400 series product family, 5. Intel. Rev.

[B39] Ionescu T. B. Schlund S. (2021). Programming cobots by voice: A human-centered, web-based approach. Procedia CIRP 97, 123–129. 10.1016/j.procir.2020.05.213

[B40] Iturrate I. Kramberger A. Sloth C. (2021). Quick setup of force-controlled industrial gluing tasks using learning from demonstration. Front. Robot. AI 8, 767878. 10.3389/frobt.2021.767878 34805294 PMC8602700

[B41] Jokesch M. Bdiwi M. Suchỳ J. (2014). Integration of vision/force robot control for transporting different shaped/colored objects from moving circular conveyor. In 2014 IEEE International Symposium on Robotic and Sensors Environments (ROSE) Proceedings (IEEE) (Timisoara, Romania: IEEE), 78–82.

[B42] Kakinuma Y. Ogawa S. Koto K. (2022). Robot polishing control with an active end effector based on macro-micro mechanism and the extended preston’s law. CIRP Ann. 71, 341–344. 10.1016/j.cirp.2022.04.074

[B43] Kalaitzakis M. Carroll S. Ambrosi A. Whitehead C. Vitzilaios N. (2020). “Experimental comparison of fiducial markers for pose estimation,” in 2020 International Conference on Unmanned Aircraft Systems (ICUAS) (Athens, Greece: IEEE), 781–789.

[B44] Kana S. Tee K.-P. Campolo D. (2021). Human–robot co-manipulation during surface tooling: A general framework based on impedance control, haptic rendering and discrete geometry. Robotics Computer-Integrated Manuf. 67, 102033. 10.1016/j.rcim.2020.102033

[B45] Karpagavalli S. Chandra E. (2016). A review on automatic speech recognition architecture and approaches. Int. J. Signal Process. Image Process. Pattern Recognit. 9, 393–404. 10.14257/ijsip.2016.9.4.34

[B46] Lambrecht J. Kleinsorge M. Rosenstrauch M. Krüger J. (2013). Spatial programming for industrial robots through task demonstration. Int. J. Adv. Robotic Syst. 10, 254. 10.5772/55640

[B47] Langmann B. Hartmann K. Loffeld O. (2012). Depth camera technology comparison and performance evaluation. ICPRAM (2), 438–444.

[B48] Lee J. (2017). A survey of robot learning from demonstrations for human-robot collaboration. 10.48550/ARXIV.1710.08789

[B49] Lei T. Huang Y. Wang H. Rong Y. (2021). Automatic weld seam tracking of tube-to-tubesheet tig welding robot with multiple sensors. J. Manuf. Process. 63, 60–69. 10.1016/j.jmapro.2020.03.047

[B50] Li H. Liu H. Cao N. Peng Y. Xie S. Luo J. (2017). Real-time rgb-d image stitching using multiple kinects for improved field of view. Int. J. Adv. Robotic Syst. 14, 172988141769556. 10.1177/1729881417695560

[B51] Lippiello V. Siciliano B. Villani L. (2005). “Eye-in-hand/eye-to-hand multi-camera visual servoing,” in Proceedings of the 44th IEEE Conference on Decision and Control (Seville, Spain: IEEE), 5354–5359.

[B52] Liu S. Wang L. Wang X. V. (2021). Sensorless haptic control for human-robot collaborative assembly. CIRP J. Manuf. Sci. Technol. 32, 132–144. 10.1016/j.cirpj.2020.11.015

[B53] Liu S. Wang L. Wang X. V. (2020). Symbiotic human-robot collaboration: Multimodal control using function blocks. Procedia CIRP 93, 1188–1193. 10.1016/j.procir.2020.03.022

[B54] Lourenço F. Araujo H. (2021). “Intel realsense sr305, d415 and l515: Experimental evaluation and comparison of depth estimation,” in Proceedings of the 16th International Joint Conference on Computer Vision, Imaging and Computer Graphics Theory and Applications (VISAPP–2021) (Online), 362 –369.

[B55] Marchand E. Uchiyama H. Spindler F. (2016). Pose estimation for augmented reality: A hands-on survey. IEEE Trans. Vis. Comput. Graph. 22, 2633–2651. 10.1109/TVCG.2015.2513408 26731768

[B56] Massa D. Callegari M. Cristalli C. (2015). Manual guidance for industrial robot programming. Industrial Robot An Int. J. 42, 457–465. 10.1108/ir-11-2014-0413

[B57] MMPose-Contributors (2020). OpenMMLab pose estimation toolbox and benchmark.

[B58] Neto P. Mendes N. (2013). Direct off-line robot programming via a common cad package. Robotics Aut. Syst. 61, 896–910. 10.1016/j.robot.2013.02.005

[B59] Park F. Martin B. (1994). Robot sensor calibration: Solving ax=xb on the Euclidean group. IEEE Trans. Rob. Autom. 10, 717–721. 10.1109/70.326576

[B60] Peng Y.-C. Carabis D. S. Wen J. T. (2018). Collaborative manipulation with multiple dual-arm robots under human guidance. Int. J. Intell. Robot. Appl. 2, 252–266. 10.1007/s41315-018-0053-y

[B61] Qiao Y. J. Liu Z. H. Hu D. X. Xu J. W. (2013). “Camera calibration method based on opencv,” in Materials engineering and automatic control II (trans tech publications ltd) (Shandong, China: Trans Tech Publications Ltd), 330, 517–520. 10.4028/www.scientific.net/AMM.330.517

[B62] Ravichandar H. Polydoros A. S. Chernova S. Billard A. (2020). Recent advances in robot learning from demonstration. Annu. Rev. Control Robot. Auton. Syst. 3, 297–330. 10.1146/annurev-control-100819-063206

[B63] Robots U. (2015). User Manual UR10/CB3 - original instructions (en), 17782. Universal Robots. Rev.

[B64] Ruan S. Wobbrock J. O. Liou K. Ng A. Landay J. (2016). Speech is 3x faster than typing for English and Mandarin text entry on mobile devices. *arXiv preprint arXiv:1608.07323* .

[B65] Sanchez-Diaz A. Zaldivar-Colado U. Pamanes-Garcia J. A. Zaldivar-Colado X. (2019). Operation of a haptic interface for offline programming of welding robots by applying a spring-damper model. Int. J. Comput. Integr. Manuf. 32, 1098–1116. 10.1080/0951192x.2019.1686177

[B66] Servi M. Mussi E. Profili A. Furferi R. Volpe Y. Governi L. (2021). Metrological characterization and comparison of d415, d455, l515 realsense devices in the close range. Sensors 21, 7770. 10.3390/s21227770 34833847 PMC8622561

[B67] Simon T. Joo H. Matthews I. Sheikh Y. (2017). “Hand keypoint detection in single images using multiview bootstrapping,” in Proceedings of the IEEE conference on Computer Vision and Pattern Recognition Hawaii, United States: CVF/IEEE.

[B68] Soares I. Petry M. Moreira A. P. (2021). Programming robots by demonstration using augmented reality. Sensors 21, 5976. 10.3390/s21175976 34502864 PMC8434657

[B69] Standard O. (2014). Mqtt version 3.1. 1. *URL* http://docs.oasis-open.org/mqtt/mqtt/v31.

[B70] Strazdas D. Hintz J. Khalifa A. Abdelrahman A. A. Hempel T. Al-Hamadi A. (2022). Robot system assistant (rosa): Towards intuitive multi-modal and multi-device human-robot interaction. Sensors 22, 923. 10.3390/s22030923 35161671 PMC8838571

[B71] Sun J. Wang Z. Zhang S. He X. Zhao H. Zhang G. (2022). “Onepose: One-shot object pose estimation without cad models,” in Proceedings of the IEEE/CVF Conference on Computer Vision and Pattern Recognition (New Orleans, United States: CVF/IEEE), 6825–6834.

[B72] Tadic V. Odry A. Burkus E. Kecskes I. Kiraly Z. Klincsik M. (2021). Painting path planning for a painting robot with a realsense depth sensor. Appl. Sci. 11, 1467. 10.3390/app11041467

[B73] Tian F. Li Z. Lv C. Liu G. (2016). Polishing pressure investigations of robot automatic polishing on curved surfaces. Int. J. Adv. Manuf. Technol. 87, 639–646. 10.1007/s00170-016-8527-2

[B74] Tirmizi A. De Cat B. Janssen K. Pane Y. Leconte P. Witters M. (2019). “User-friendly programming of flexible assembly applications with collaborative robots,” in 2019 20th International Conference on Research and Education in Mechatronics (REM) (Wels, Australia: IEEE), 1–7.

[B75] Tsai R. Lenz R. (1989). A new technique for fully autonomous and efficient 3d robotics hand/eye calibration. IEEE Trans. Rob. Autom. 5, 345–358. 10.1109/70.34770

[B76] Tykal M. Montebelli A. Kyrki V. (2016). “Incrementally assisted kinesthetic teaching for programming by demonstration,” in 2016 11th ACM/IEEE International Conference on Human-Robot Interaction (HRI) (Christchurch, New Zealand: IEEE), 205–212.

[B77] van Delden S. Umrysh M. Rosario C. Hess G. (2012). Pick-and-place application development using voice and visual commands. Industrial Robot An Int. J. 39, 592–600. 10.1108/01439911211268796

[B78] Villani V. Pini F. Leali F. Secchi C. (2018). Survey on human–robot collaboration in industrial settings: Safety, intuitive interfaces and applications. Mechatronics 55, 248–266. 10.1016/j.mechatronics.2018.02.009

[B79] Voice INTER connect GmbH (2022). vicCONTROL industrial version 6.3.0 - User guide - phytec Voice Control Kits (phyBOARD®-Mira). voice INTER connect GmbH, 1. Rev.

[B80] Wrede S. Emmerich C. Grünberg R. Nordmann A. Swadzba A. Steil J. (2013). A user study on kinesthetic teaching of redundant robots in task and configuration space. J. Hum. Robot. Interact. 2, 56–81. 10.5898/jhri.2.1.wrede

[B81] Xiang Y. Schmidt T. Narayanan V. Fox D. (2017). Posecnn: A convolutional neural network for 6d object pose estimation in cluttered scenes. *arXiv preprint arXiv:1711.00199* .

[B82] Yang C. Luo J. Liu C. Li M. Dai S.-L. (2018a). Haptics electromyography perception and learning enhanced intelligence for teleoperated robot. IEEE Trans. Autom. Sci. Eng. 16, 1512–1521. 10.1109/tase.2018.2874454

[B83] Yang L. Li E. Long T. Fan J. Liang Z. (2018b). A novel 3-d path extraction method for arc welding robot based on stereo structured light sensor. IEEE Sens. J. 19, 763–773. 10.1109/jsen.2018.2877976

[B84] Zakiev A. Tsoy T. Shabalina K. Magid E. Saha S. K. (2020). “Virtual experiments on aruco and apriltag systems comparison for fiducial marker rotation resistance under noisy sensory data,” in 2020 International Joint Conference on Neural Networks (IJCNN) (Glassgow, United Kingdom: IEEE), 1–6.

[B85] Zhang B. Wu J. Wang L. Yu Z. (2020a). Accurate dynamic modeling and control parameters design of an industrial hybrid spray-painting robot. Robotics Computer-Integrated Manuf. 63, 101923. 10.1016/j.rcim.2019.101923

[B86] Zhang F. Bazarevsky V. Vakunov A. Tkachenka A. Sung G. Chang C.-L. (2020b). Mediapipe hands: On-device real-time hand tracking. 10.48550/ARXIV.2006.10214

[B87] Zhang H.-D. Liu S.-B. Lei Q.-J. He Y. Yang Y. Bai Y. (2020c). Robot programming by demonstration: A novel system for robot trajectory programming based on robot operating system. Adv. Manuf. 8, 216–229. 10.1007/s40436-020-00303-4

[B88] Zhang S. Wang S. Jing F. Tan M. (2019). A sensorless hand guiding scheme based on model identification and control for industrial robot. IEEE Trans. Ind. Inf. 15, 5204–5213. 10.1109/tii.2019.2900119

[B89] Zhou H. Ma S. Wang G. Deng Y. Liu Z. (2021). A hybrid control strategy for grinding and polishing robot based on adaptive impedance control. Adv. Mech. Eng. 13, 168781402110040. 10.1177/16878140211004034

[B90] Zirkelbach C. Krause A. Hasselbring W. (2019). Modularization of research software for collaborative open source development. *arXiv preprint arXiv:1907.05663* .

